# Temporal order of RNase IIIb and loss-of-function mutations during development determines phenotype in pleuropulmonary blastoma / *DICER1* syndrome: a unique variant of the two-hit tumor suppression model

**DOI:** 10.12688/f1000research.6746.2

**Published:** 2018-01-12

**Authors:** Mark Brenneman, Amanda Field, Jiandong Yang, Gretchen Williams, Leslie Doros, Christopher Rossi, Kris Ann Schultz, Avi Rosenberg, Jennifer Ivanovich, Joyce Turner, Heather Gordish-Dressman, Douglas Stewart, Weiying Yu, Anne Harris, Peter Schoettler, Paul Goodfellow, Louis Dehner, Yoav Messinger, D. Ashley Hill

**Affiliations:** 1 Division of Pathology, Children’s Research Institute, Children's National Medical Center and the George Washington University School of Medicine & Health Sciences, Washington, DC, 20010, USA; 2 Center for Genetic Medicine Research, Children’s Research Institute, Children's National Medical Center and the George Washington University School of Medicine & Health Sciences, Washington, DC, 20010, USA; 3 International Pleuropulmonary Blastoma Registry, Children’s Hospitals and Clinics of Minnesota, Minneapolis, MN, 55404, USA; 4 Department of Oncology, Children’s Hospitals and Clinics of Minnesota, Minneapolis, MN, 55404, USA; 5 Division of Oncology, Children’s Research Institute, Children's National Medical Center and the George Washington University School of Medicine & Health Sciences, Washington, DC, 20010, USA; 6 Department of Pathology, Johns Hopkins School of Medicine, Baltimore, MD, 21205, USA; 7 Department of Surgery, Washington University Medical Center, St. Louis, MO, 63110, USA; 8 Division of Genetics, Children’s Research Institute, Children's National Medical Center and the George Washington University School of Medicine & Health Sciences, Washington, DC, 20010, USA; 9 Department of Integrative Systems Biology, George Washington University School of Medicine & Health Sciences, Washington, DC, 20010, USA; 10 Clinical Genetics Branch, Division of Cancer Epidemiology and Genetics, National Cancer Institute, NIH, Rockville, MD, 20892, USA; 11 College of Medicine, The Ohio State University, Columbus, OH, 43210, USA; 12 Lauren V. Ackerman Laboratory of Surgical Pathology, Washington University Medical Center, St. Louis, MO, 63110, USA

**Keywords:** DICER1 truncation, PPB, Pleuropulmonary blastoma, Mosaicism, Paediatric cancer, RNAse IIIb

## Abstract

Pleuropulmonary blastoma (PPB) is the most frequent pediatric lung tumor and often the first indication of a pleiotropic cancer predisposition, *DICER1* syndrome, comprising a range of other individually rare, benign and malignant tumors of childhood and early adulthood. The genetics of *DICER1*-associated tumorigenesis are unusual in that tumors typically bear neomorphic missense mutations at one of five specific “hotspot” codons within the RNase IIIb domain of *DICER 1*, combined with complete loss of function (LOF) in the other allele. We analyzed a cohort of 124 PPB children for predisposing *DICER1* mutations and sought correlations with clinical phenotypes. Over 70% have inherited or *de novo* germline LOF mutations, most of which truncate the *DICER1* open reading frame. We identified a minority of patients who have no germline mutation, but are instead mosaic for predisposing *DICER1* mutations. Mosaicism for RNase IIIb domain hotspot mutations defines a special category of *DICER1* syndrome patients, clinically distinguished from those with germline or mosaic LOF mutations by earlier onsets and numerous discrete foci of neoplastic disease involving multiple syndromic organ sites. A final category of PBB patients lack predisposing germline or mosaic mutations and have sporadic (rather than syndromic) disease limited to a single PPB tumor bearing tumor-specific RNase IIIb and LOF mutations. We propose that acquisition of a neomorphic RNase IIIb domain mutation is the rate limiting event in *DICER1*-associated **tumorigenesis, and that distinct clinical phenotypes associated with mutational categories reflect the temporal order in which LOF and RNase IIIb domain mutations are acquired during development.

## Introduction

Pleuropulmonary blastoma (PPB) is the most common primary lung cancer of childhood (OMIM #601200) ^1, 2^. Early PPB (type I) presents as lung cysts that are at risk for transformation into high grade sarcomas, which may have both cystic and solid components (PPB type II) or be entirely solid (PPB type III) ^2, 3^. Not all PPB type I cysts progress to sarcoma; those that do not are designated type Ir (regressed) ^1, 3^. The genetic and epigenetic events responsible for initiation of cyst formation and subsequent progression to sarcoma are just beginning to be understood ^3– 6^. PPB is pathognomonic for a cancer predisposition syndrome that features a range of other benign and malignant neoplasms including ovarian Sertoli-Leydig cell tumor (SLCT), cystic nephroma (CN) and renal sarcoma or Wilms tumor, nodular hyperplasia and carcinoma of the thyroid gland, nasal chondromesenchymal hamartoma (NCMH), embryonal rhabdomyosarcoma (ERMS), pituitary blastoma and pineoblastoma ^2, 4, 7– 30^. Although most syndromic neoplasias arise in childhood or adolescence, occasional onsets in adulthood have been seen for some tumor types, notably SLCT ^27^. We previously identified inherited loss of function (LOF) mutations in *DICER1* (OMIM #606241) as the major genetic factor in this syndrome ^4^. *DICER1* syndrome thus became the first cancer predisposition associated with a systemic defect in microRNA (miRNA) processing.

The *DICER1* gene encodes an RNase III-family endonuclease that cleaves precursor microRNAs (pre-miRNA) into active miRNA ^31, 32^. Sequencing studies of syndromic tumors have revealed biallelic, compound mutations of *DICER1* ^6, 11, 15, 21, 26, 28– 30, 33– 35^. Generally, one allele (often germline) bears a nonsense or frame-shift mutation predicted to cause full loss of function (LOF), and one allele bears a missense mutation in the *DICER1* RNase IIIb domain. Biallelic LOF mutations have not been identified in PPB, suggesting that retention of some miRNA processing function is usually required for tumor survival ^6, 35^. RNase IIIb missense mutations in *DICER1* syndrome tumors affect five "hotspot" codons that encode key amino acids in the metal-binding catalytic cleft of the nuclease domain: E1705, D1709, G1809, D1810 and E1813 ^6, 26, 29, 30, 33– 35^. Amino acid substitutions at these positions cause neomorphic *DICER1* function in miRNA processing, such that cleavage of mature 5p miRNAs from the 5’ end of pre-miRNA hairpin structures fails, while mature 3p miRNAs continue to be cleaved from the 3’ end normally ^6, 26, 33, 35, 36^. The high overall ratio of 5p to 3p mature miRNAs seen in normal tissues is essentially inverted in *DICER1* tumors, suggesting that uncleaved 5p miRNAs are rapidly degraded ^6^. Depletion of 5p miRNAs alters expression of numerous downstream target mRNAs across the exome, including some critical for embryogenesis or tumor suppression ^33, 36^. The pleiotropic nature of *DICER1* syndromic disease likely reflects the diverse array of genes regulated by miRNAs during organ development and in differentiated tissues.

Clinical features of *DICER1* syndrome are highly variable with regard to age at first occurrence of neoplastic disease, the number of discrete foci of disease that develop over time, and the specific organ sites involved. As a step toward understanding the basis of clinical variability, we explored the spectrum of predisposing *DICER1* mutations in a large cohort of PPB/ *DICER1* syndrome patients. Correlation of genotypes with clinical features revealed a distinctive phenotype of early onsets and extensive, multifocal disease in patients who are mosaic for hotspot missense mutations in the RNase IIIb domain. We propose that the extreme phenotypes of this patient group are attributable to the order in which allelic *DICER1* mutations were acquired during development, *i.e.,* an RNase IIIb hotspot missense mutation acquired early in embryogenesis and subsequently unmasked by LOF mutations or loss of the second allele. Understanding how the interplay of RNase IIIb missense and LOF mutations influences the expression of syndromic neoplasias can aid diagnosis at early stages, and improve genetic evaluation and counseling for families with *DICER1* syndrome.

## Subjects and methods

### Patients and specimens

PPB patients (n = 124) and family members were ascertained through the International PPB Registry (IPPBR). Inclusion into this study required a pathologic diagnosis of PPB verified by central review (LPD, DAH). All subjects gave written consent for molecular and family history studies, as approved by the Human Research Protection Offices at Washington University in St. Louis (HSC#04-1154), Children's Hospitals and Clinics of Minnesota (IRB#98107), and Children's National Medical Center (IRB#4603; Pro0315). For families with more than one affected member, only data from the initial proband is included. Medical history and biological samples were collected and prepared for analysis as previously described ^4, 30^. Tumor tissue was available for sequencing from a subset of patients. For two of these cases, DNA was isolated from unstained tissue on glass slides using the Pinpoint Slide DNA Isolation System (Zymo, Irvine, CA).

### Definition of “disease foci”

Clinical data were abstracted from medical records and imaging studies. All children had pathologic confirmation of PPB. The following lesions were defined as evidence of syndromic disease and scored as disease foci: lung cysts, kidney cysts, cystic nephroma, Wilms tumor, thyroid nodules or carcinoma, ovarian Sertoli-Leydig cell tumor (SLCT), nasal chondromesenchymal hamartoma (NCMH), embryonal rhabdomyosarcoma (ERMS) of the uterine cervix or urinary bladder, ciliary body meduloepithelioma (CBME), pineoblastoma, pituitary blastoma and juvenile-type polyps of the small intestine. Lung cysts that were distinctly separate (in different lobes or anatomically separated within the same lobe) and renal cysts in contralateral kidneys were scored as individual disease foci ( Table 1).

### Mutation testing

Initial sequencing of blood and saliva DNA samples was by standard Sanger methods described previously ^4^ or by a commercial laboratory (Ambry Genetics, Aliso Viejo, CA). Low-frequency variants were detected and quantified by targeted next-generation sequencing (NGS) using a custom multiplex PCR panel for *DICER1* coding regions (Ion Torrent Ampliseq, Life Technologies, Grand Island, NY, USA) ( Table S1) ^30^. NGS was performed on an Ion Torrent 318 v2 chip (ION PGM Sequencing 200 kit v2, Life Technologies) with an average of 6 samples per chip, to achieve an average depth of coverage of 3000 filtered reads. Signal processing, mapping and quality control were performed with Torrent Suite software v.4.0.2 (Life Technologies). Variant calls were made using the Torrent Variant Caller Plugin v.4.0, with somatic low stringency mutation workflow and default settings. BAM files of raw reads were reviewed using Integrative Genomics Viewer v2.3 ^37, 38^.

### Annotation of sequence variants and the spectrum of possible mutations

*DICER1* sequence variants were annotated with Alamut Batch software (Interactive Biosoftware, Rouen, France), with reference to *DICER1* transcript record NM_177438.2. Nonsense, frameshift and canonical splice-site mutations were considered loss of function (LOF). Missense variants affecting codons 1705, 1709, 1809, 1810 and 1813 in the RNase IIIb domain were classified as “hotspot” mutations. For variants assayed by NGS, allele frequencies were calculated from filtered read counts. The SIFT and PROVEAN algorithms were used to assess potential significance of novel missense mutations ^39– 43^. All variants identified were deposited into ClinVar (accession numbers SCV000195560-SCV000195643). The numbers of possible single-nucleotide changes that can produce amino acid substitutions at the five hotspot codons or nonsense mutations anywhere in the *DICER1* open reading frame, or disrupt canonical splice sites, were compiled from *DICER1* transcript record NM_177438.2 and genomic record NG_016311.1.

### NanoString genomic copy number assay of germline DNA

In a few cases, NanoString Copy Number Assay at was used to screen for *DICER1* exonic deletions in genomic DNA extracted from blood. It was not used to assess locus copy number in formalin-fixed tumor specimens. Molecular probes for the *DICER1* locus were developed in collaboration with NanoString Technologies, Inc., Seattle, WA ( Table S2). Genomic DNA was fragmented and hybridized using the nCounter Prep Station, and hybridization signals quantified using the nCounter Digital Analyzer, according to NanoString’s recommendations. Preliminary analysis and quality control of the data were performed using nSolver Analysis Software version 1.1 (NanoString) with default copy number variation (CNV) analysis settings. CNVs were confirmed with high-density CNV array hybridization in a commercial laboratory (Prevention Genetics, Marshfield, WI).

### Statistical analyses

The number of disease foci per patient and the age at *DICER1* syndrome diagnosis were compared between mutation categories using nonparametric tests, due to the skewness of both clinical features and to the unbalanced sample sizes. Kruskal-Wallis tests were used to compare medians among the four mutation categories. Where a significant overall association was found, pair-wise post-hoc Wilcoxon rank sum tests were used to compare medians, and resulting p-values adjusted for multiple comparisons using the Sidak method. A p-value of 0.05 was considered statically significant and all analyses were performed using Stata V13 (College Station, TX).

## Results

### Most predisposing *DICER1* mutations are inherited loss of function (LOF) mutations

**Figure 1.**
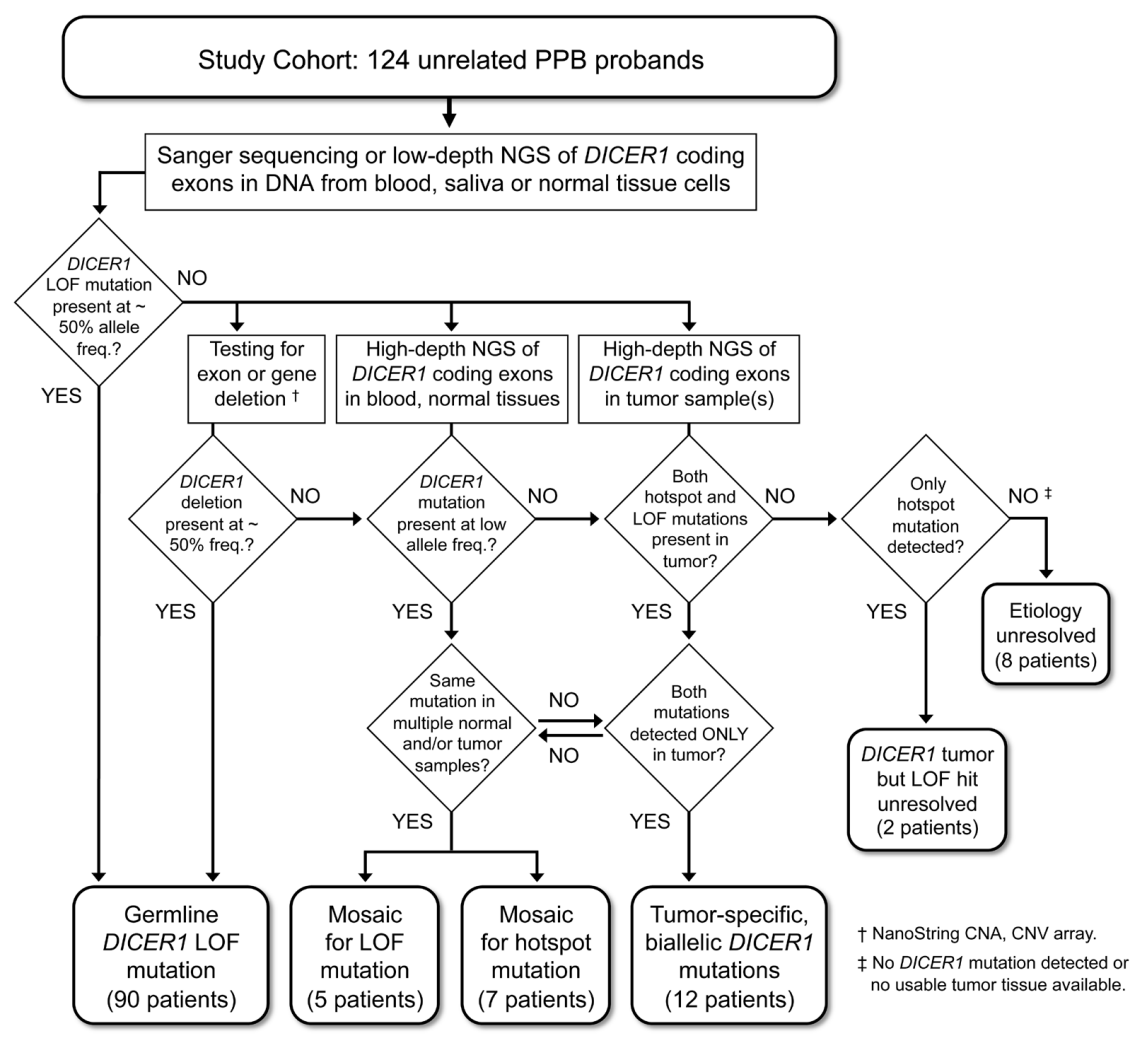
Study design – Detection and categorization of *DICER1* mutations in PPB probands. A cohort of 124 children diagnosed with pleuropulmonary blastoma (PPB) was screened for predisposing *DICER1* mutations by targeted Sanger sequencing and/or low-depth, next-generation sequencing (NGS) of DNA amplified from peripheral blood cells, saliva (buccal cells) or non-neoplastic surgical specimens. Sequenced PCR amplicons covered the 26 coding exons of the *DICER1* open reading frame and flanking splice signals. *DICER1* coding sequence or splice site mutations detected at approximately heterozygous frequency in blood or normal tissue cells were categorized as germline mutations. For patients in whom screening revealed no germline mutation, blood and/or normal tissues were analyzed for the presence of intragenic deletions or larger genomic alterations using NanoString copy number assay and CNV array, and for coding or splice site mutations present at low allele frequencies using high-depth NGS on the Ion Torrent platform. Wherever possible, matched tumor specimens were also sequenced on the Ion Torrent platform. *DICER1* mutations detected in tumor samples and at sub-heterozygous frequencies in blood or other normal tissue samples were categorized as mosaic mutations. RNase IIIb hotspot mutations detected in primary tumors of multiple organs were also categorized as mosaic mutations, even if they were not conclusively identified in blood or other normal tissues. Patients for whom both LOF and hotspot mutations were identified in a single tumor, but not found in blood or normal tissue samples, were categorized as having tumor-specific, biallelic *DICER1* mutations. Cases of this last kind are considered sporadic PPB, not DICER1 syndrome.

Our overall approach to detecting and categorizing predisposing *DICER1* mutations in PPB children is shown schematically in Figure 1. We identified germline, heterozygous *DICER1* mutations in 90 of the 124 probands in our cohort (72.6%; Table 1, Table S3). Nearly all (89) were detected by Sanger sequencing of exonic PCR amplicons. For one child in whom no mutation was detected by Sanger sequencing, blood DNA was probed by NanoString hybridization, which indicated deletion of one copy of exon 24. High-density CNV array hybridization was used to confirm a heterozygous deletion of ~ 1.1 kb, comprising all of exon 24 and parts of the flanking introns (c.5096-498_5364+356del). Paternal DNA was positive for the deletion, which was anticipated as this child has an uncle with CN. Only one previous instance of a large, intragenic deletion as a germline *DICER1* mutation has been reported, which suggests such mutations are very rare ^44^. The actual prevalence of large deletions is difficult to estimate because they are not readily detected by the targeted sequencing strategies applied for mutation screening in this study and most others.

**Table 1.** Clinical and Pathologic Features by Predisposing *DICER1* Mutation Category.

	Germline LOF mutations	Mosaic mutations		Tumor- specific mutations
		Loss of function	RNase IIIb hotspot	
Number of patients	90	5	7	12
Sex distribution				
Male	44	3	4	10
Female	46	2	3	2
Age at first diagnosis, months ^a^				
Median (range)	35 (0–227)	25 (12–46)	12 (0–18)	33 (24–139)
Mean (standard deviation)	36 (31)	27 (12)	11 (6)	42 (31)
P-value, vs. germline group ^d^	–	0.97	0.0161	0.99
Disease foci distribution				
Lung - cysts, PPB	90	5	7	12
Kidney - cysts, cystic nephroma	12	0	6	0
Kidney - Wilms tumor	1	0	0	0
Thyroid - nodular hyperplasia	4	0	2	0
Thyroid - cancer	4	0	1	0
Nasal cavity - NCMH	5	0	2	0
Ovary - Sertoli-Leydig cell tumor	3	0	2	0
Uterine cervix - ERMS	4	0	0	0
Urinary bladder - ERMS	2	0	0	0
Pineoblastoma	1	0	1	0
Ciliary body medulloepithelioma	1	0	1	0
Small intestine - juvenile polyps	0	0	4	0
Small intestine - juvenile polyps	0	0	4	0
Pelvic sarcoma	0	0	1	0
PPB type distribution				
Type Ir	9	0	5 ^c^	0
Type I	25	2	1	1
Type II	31	1	2 ^c^	6
Type III	25	2	0	5
Number of disease foci per patient ^b^				
Median (range)	2 (1–6)	2 (1–2)	13 (9–24)	1 (1–1)
Mean (standard deviation)	1.8 (1.0)	1.6 (0.5)	15 (6.4)	1 (0.0)
P-value, vs. germline group ^d^	–	0.99	0.0001	0.0072
Survival, number of patients (months)				
Alive (median age at present)	80 (100)	3 (46)	6 (87)	10 (85)
Deceased (median age at death)	10 (60.5)	2 (64.5)	1(132)	2 (57)

Abbreviations: PPB pleuropulmonary blastoma; NCMH nasal chondromesenchymal hamartoma; ERMS embryonal rhabdomyosarcoma.

a. Age at first clinical presentation with PPB or other *DICER1* syndrome pathology.

b. Total number of discrete disease foci, as defined in Subjects and Methods.

c. Two patients with both type Ir and type II PPB.

d. Medians compared using a Kruskal-Wallis test; *post-hoc* pair-wise tests adjusted for multiple comparisons.

**Figure 2.**
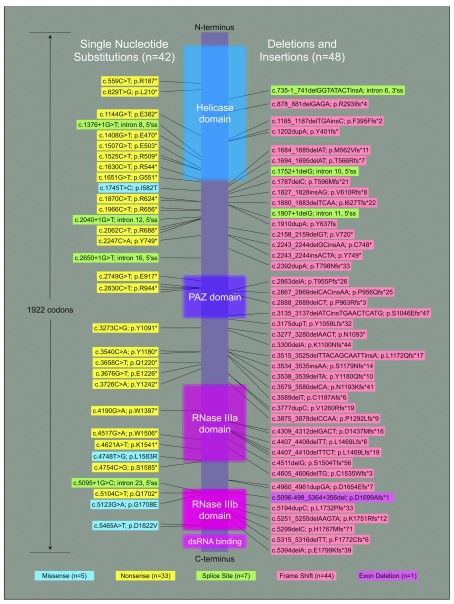
The spectrum of predisposing loss-of-function mutations in PPB/ *DICER1* syndrome. A linear schematic of the *DICER1* open reading frame is shown with annotated functional domains represented to scale. Sequence changes identified as inherited or *de novo* germline mutations in 90 PPB/ *DICER1* syndrome patients are indicated by position along the coding sequence. Mutations linked to the schematic by two, three or four fine lines are those discovered in a corresponding number of individuals from unique families.

The spectrum of germline mutations is dominated by truncating, LOF mutations ( Figure 2). These are mainly single-nucleotide substitutions that produce new stop codons (33 cases, 37%) and small insertions or deletions (indels) within exons that shift reading frame (44 cases, 49%). Seven mutations of consensus splice sites occur in our cohort; of which six are predicted to cause exon skipping during transcript splicing with resulting frameshift. The remaining splice site mutation, c.1752+1delG, is at the 5’ end of intron 10. Skipping of exon 10 would cause in-frame deletion of 81 amino acids near the end of the helicase domain. In all, 84 of 90 germline *DICER1* mutations discovered in patients (93%) truncate the open reading frame before the end of the critical RNase IIIb domain, and are thus predicted to result in complete loss of DICER1 protein function even if the message escapes nonsense-mediated decay. Six non-truncating germline mutations were identified, including the intron 10 splice site mutation described above and five non-hotspot missense changes: I582T, L1583R and G1708E (each seen once) and D1822V (identified in two patients) ( Table S4). The I582T substitution is at the distal end of the helicase domain ( Figure 2), the role of which is unclear. L1583R is within the RNase IIIa domain and segregates with disease in a family ^4^. The G1708E and D1822V mutations both fall within the RNase IIIb domain, near the metal-binding catalytic site. These latter two missense mutations are predicted to compromise protein function by the SIFT and PROVEAN algorithms ( Table S4), but their precise functional significance in DICER1 is unknown ^39– 43^.

DNA was available from both parents for 77 children with germline mutations, and Sanger sequencing of parental DNA was sufficient to confirm 67 of the mutations (87%) as inherited. Mutations in the ten patients whose parents had no *DICER1* mutation detected by Sanger sequencing were provisionally considered *de novo*. To confirm this, targeted next generation sequencing (NGS) was performed in eight of the ten triads, yielding mutant allele frequencies between 42.0% and 57.1% in the probands but no conclusive evidence of the variants in parental blood. For some triads, a few reads matching the proband mutation were obtained from one or both parents, and in one triad, (study ID# 59) mutant reads were obtained in both parents at frequencies slightly above the predicted error rates for the sequencing platform ( Table S5). We interpret this as marginal evidence at best for parental mosaicism. None of the 10 probands with apparent *de novo* mutations had known family members with syndromic disease. There were no statistically significant differences between *de-novo* and inherited germline LOF patients with respect to age at onset, numbers of disease foci or survival.

Penetrance of familial *DICER1* LOF mutations was far from complete. Of the 67 families in this cohort with segregating LOF mutations, 29 include parents or siblings who are confirmed as mutation carriers but have no history of syndromic disease ( Table S6). True penetrance is difficult to estimate because we have limited knowledge of how many germline *DICER1* mutation carriers are phenotypically normal, as only a subset with overtly affected family members have been ascertained. Moreover, subclinical disease is common. Preliminary data from an ongoing NCI-sponsored *DICER1* family history study indicate that ~ 87% of otherwise asymptomatic individuals with confirmed *DICER1* mutations have thyroid nodules detectable by ultrasound and ~ 43% have lung cysts detectable by CT scan (D.R. Stewart and L. Doros, unpublished).

Among children with germline LOF mutations, age at first diagnosis of PPB or other syndromic disease was typically one to five years (70 of 90 patients), but this ranged from diagnosis within days of birth to as late as eighteen years. The most frequent syndromic condition after PPB was cystic nephroma, followed by thyroid disease (nodular hyperplasia or carcinoma), nasal chondromesenchymal hamartomas and embryonal rhabdomyosarcomas ( Table 1, Table S6). The number of discrete disease foci per patient ranged as high as five or six (in two patients), but the majority of children in this group had experienced no more than two at the time of their most recent exam, and nearly half had only a single PPB tumor. None of the six patients with non-truncating germline mutations had unusual clinical features and as a group they were not distinguishable from patients with truncating mutations. Table S7 provides data on somatic hotspot mutations identified in all available tumors of PPB children.

### Approximately 10% of predisposing *DICER1* mutations are mosaic rather than germline

We and others have previously described biallelic *DICER1* mutations in tumors of children who apparently have no germline mutation, inherited or *de novo* ^6, 14, 35^. Because PPB children are typically so young when affected, we hypothesized that at least some cases of this kind reflect mosaicism, *i.e.,* a mutation present in some but not all cells of the body, because it occurred during post-zygotic embryonic development rather than being present in the zygote (as a germline mutation would be). To explore this possibility, we performed targeted, high-depth NGS of *DICER1* coding exons in DNA from blood and/or other normal tissues of children who had tested negative for germline mutation by Sanger sequencing, and in matched samples of tumor tissue where available. We categorized a *DICER1* mutation detected by NGS as mosaic when the following criteria were met: *i.* The mutation was evidently not a constitutional, germline allele because it was present at sub-heterozygous frequency (arbitrarily taken as below 35% of reads) in peripheral blood and/or other normal tissue samples. *ii.* The mutation was evidently not specific to a tumor, because the same mutant allele was detected in one or more normal, non-neoplastic tissue samples, OR, the same mutant allele was detected in multiple primary tumors arising in different organs ( Figure 1). We identified twelve children with predisposing mosaicism for either LOF or RNase IIIb hotspot mutations ( Table 1).

Mosaic LOF mutations were detected in five children, at frequencies that ranged from 1.1% to 17.2% of allelic reads in DNA from blood, saliva or normal fibroblasts ( Table S8). For three of these children, archival PPB tumor tissue was available, and in each the LOF mutation was present, as was an RNase IIIb domain hotspot mutation. Two of the five children with mosaic LOF mutations had a single focus of disease in a lung. The other three children each had two foci of disease, also restricted to the lungs. It might be anticipated that children bearing mosaic LOF mutations tend to have fewer disease foci than those with germline LOF mutations because the number of cells at risk for second hits is generally lower. No statistically significant difference of this kind can be discerned from the five mosaic LOF children in our cohort, but notably, none have developed syndromic tumors other than PPB. As this was not a population study, we cannot estimate how many persons with mosaic LOF mutations are asymptomatic but, by analogy to the low penetrance of familial LOF mutations, it could be a large proportion.

 Seven children in the cohort harbored mosaic RNase IIIb domain hotspot missense mutations, detected in multiple primary neoplasms and/or non-neoplastic tissues ( Table 2). None had family members with features of *DICER1* syndrome, and the RNase IIIb hotspot mutations found in probands were not detected in parental blood, consistent with a postzygotic origin. NGS of tumor tissues from these children also identified somatic LOF mutations or evidence of allele loss in all specimens, with the caveat that allele loss can be difficult to establish in tumor specimens of low purity, prticularly non-malignant / pre-malignant lesions that comprise a mixture of neoplastic and non-neoplastic cell types ( *e.g.*, PPB Type Ir, CN and NCMH). For one mosaic hotspot patient, study ID# 105, specimens of a thyroid carcinoma and two separate ovarian Sertoli-Leydig cell tumors (SLCT) were available for NGS. The thyroid carcinoma and one SLCT had apparently lost the second *DICER1* allele, but the other SLCT had instead sustained a frameshift mutation. Similarly for study ID# 104, specimens of a cystic nephroma and two separate SLCTs were available. One SLCT had clearly lost its wild-type *DICER1* allele, but the cystic nephroma and the second SLCT carried two different frameshift mutations ( Table 2). These results are consistent with underlying mosaicism for the RNase IIIb hotspot mutation and subsequent acquisition of independent LOF mutations or allele loss in each tumor site.

**Table 2.** Sequence Results from Children with *DICER1* Mosaic RNase IIIb Mutations.

Study ID	Tissue source	Tumor purity ^a^	RNase IIIb domain hotspot mutation	Hotspot allele freq. ^b^ (variant/total reads)	Loss of function mutation	LOF allele freq. ^b^ (variant/total reads)
101	Blood	–	c.5126A>G; p.D1709G	Sanger ^c^ (NR)	ND	–
	Normal lymph node	–	“	15.2% (10/66)	ND	–
102	Blood	–	c.5125G>A; p.D1709N	4.61% (22/477)	ND	–
	Brain, PPB metastasis	30%	“	51.0% (213/418)	Allele loss	–
103	Blood	–	c.5125G>A; p.D1709N	0.28% (18/6413)	ND	–
	Kidney, CN	40%	“	14.9% (174/1172)	c.1129G>A; p.V377I	3.1% (5/159)
	Lung, PPB Type IR	20%	“	16.2% (30/185)	c.1200G>A; p.W400*	4.1% (11/141)
	Small intestine, polyp	25%	“	17.5% (65/371)	c.96G>A; p.W32*	3% (13/431)
104	Blood	–	c.5428G>T; p.D1810Y	0.21% (13/6217)	ND	–
	Normal fallopian tube	–	“	7.19% (141/1961)	ND	–
	Lung, PPB Type IR	20%	“	27.8% (193/694)	ND	–
	Kidney, CN	20%	“	29.2% (64/219)	c.1711delT; p.S571Vfs*16	21.8% (73/192)
	Ovary (right), SLCT	34%	“	34.2% (684/1988)	c.1775delA; p.K592Mfs*15	36.2% (721/1993)
	Ovary (left), SLCT	95%	“	92.4% (1837/1988)	Allele loss	–
105	Blood	–	c.5437G>C; p.E1813Q	0.04% (1/2450) ^d^	ND	–
	Nasal cavity, NCMH	20%	“	29.3% (579/1977)	ND	–
	Thyroid, follicular Ca	60%	“	66.6% (289/434)	Allele loss	–
	Ovary (right), SLCT	75%	“	76.8% (750/976)	Allele loss	–
	Ovary (left), SLCT	25%	“	31.8% (624/1962)	c.4626delC; p.Q1542Hfs*18	21.7% (430/1984)
120 (de Kock ^45^ case 4) ^e^	Blood	–	ND	–	ND	–
	Reactive lung	–	c.5425G>A; p.G1809R	1% (36/3442)	ND	–
	Lung, PPB type II	NR	“	37% (1455/3972)	c.1966C>T; p.R656*	NR
123	Blood	–	ND	–	ND	–
	Normal ureter	–	c.5113G>A; p.E1705K	13% (19/148)	ND	–
	Lung, PPB type I	20	“	24% (46/192)	Allele loss	–
	Kidney, CN	25	“	35% (33/94)	Allele loss	–
Klein ^46^ case 1	Blood	–	c.5138A>T; p.D1713V	21% (NR)	ND	–
	Normal kidney	–	“	35% (NR)	ND	–
	Wilms tumor	NR	“	37% (NR)	c.1304C>T; p.P453L	variable
Klein ^46^ case 2	Blood	–	c.5125G>T; p.D1709Y	28% (NR)	ND	–
	Normal kidney	–	“	35% (NR)	ND	–
	Wilms tumor	NR	“	47% (NR)	ND	–
De Kock ^11^ case 12	Blood	–	c.5125G>C; p.D1709H	Sanger ^c^ (NR)	ND	–
	Pituitary blastoma	NR	“	Sanger ^c^ (NR)	Allele loss	–

Abbreviations: Ca carcinoma; CN cystic nephroma; LOF loss of function; NCMH nasal chondromesenchymal hamartoma; ND none detected; NR not reported; PPB pleuropulmonary blastoma; SLCT Sertoli-Leydig cell tumor.

a. Percent tumor cells in specimen, estimated visually by microscopy in tumor sections.

b. Allele frequency estimates were derived from NGS read counts in this study. In the two cases reported by Klein *et al.*, allele frequencies were determined by pyrosequencing assays.

c. Hotspot allele detected by Sanger sequencing only; no NGS performed.

d. Variant allele frequency below estimated error rate for base substitutions (0.07%) with Ion Torrent using 200 bp sequencing kit ^47^.

e. *Note addded in revision:* Sequence data shown for study ID# 120 was published by de Kock *et al.* ^45^. We concur in their conclusion of mosaicism.

### Mosaic RNase IIIb hotspot mutations are associated with early-onset, multifocal disease

**Figure 3.**
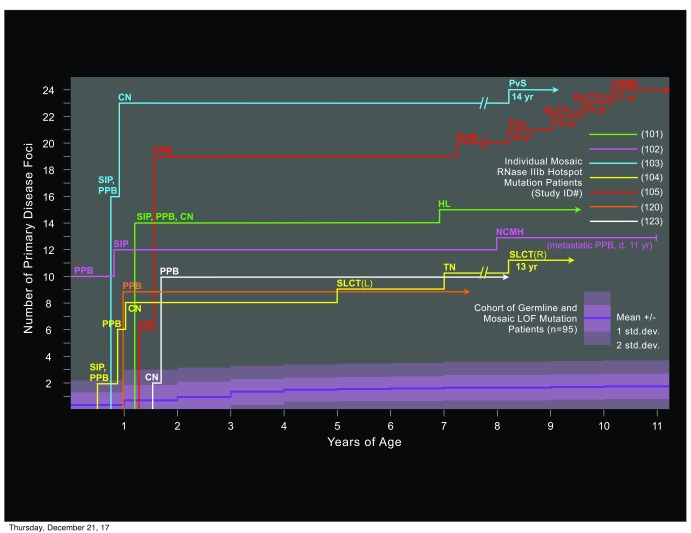
Numbers and types of disease foci in *DICER1* syndrome patients with mosaic RNase IIIb domain hotspot mutations. For each of the seven mosaic hotspot children identified in this study, an individual timeline indicates numbers of discrete foci of neoplastic disease and their histopathological types, graphed with respect to patient age at diagnosis. Across the lower portion of the chart, a single aggregate timeline (dark violet) represents the mean number of disease foci for all PPB/ *DICER1* syndrome patients with predisposing loss of function (LOF) mutations identified in this study, graphed with respect to patient age at diagnosis. The shaded areas (in lighter violet) surrounding the timeline for LOF mutation patients indicates one and two standard deviations above and below the mean. The range of foci number among all LOF mutation patients was 0 to 6 in all years of age represented (not shown). Abbreviations: CN cystic nephroma; CBME ciliary body medulloepithelioma (eye); NCMH nasal chondromesenchymal hamartoma; PPB pleuropulmonary blastoma; PinB pineoblastoma; PvS pelvic sarcoma; SIP small intestinal polyp(s); SLCT Sertoli-Leydig cell tumor (ovary); TCa thyroid carcinoma; TN thyroid nodule(s).

The seven children with mosaic RNase IIIb domain hotspot mutations shared unusual clinical features. All were diagnosed with *DICER1* syndrome early; within 15 months of birth. All presented with multiple cysts of the lungs and/or kidneys, which were accompanied or followed in all cases by multiple *DICER1* syndromic tumors ( Figure 3). Four of the seven had CN as well as PPB. Other tumors included SLCT, thyroid nodular hyperplasia or carcinoma, NCMH, ciliary body medulloepithelioma, one pineoblastoma and one pelvic sarcoma with histopathologic features similar to those of PPB. In addition, four children had juvenile-type polyps of the small intestine, discovered upon surgical intervention for intestinal intussusception. Total numbers of discrete disease foci per patient were extraordinarily high, ranging from a minimum of 9 or 10 to as many as 24. Despite the small number of patients in this group, statistical analysis confirms clinical impressions that they are distinct from those with predisposing LOF mutations. Mean age at first DICER1 syndrome diagnosis was significantly earlier, and both mean and median numbers of disease foci are significantly greater in children with mosaic RNase IIIb mutations ( Table 1). The association with juvenile-type intestinal polyps and intussusception may be a novel feature of children with mosaic RNase IIIb hotspot mutations, as no diagnoses of intestinal polyps were reported in children with germline or mosaic *DICER1* LOF mutations.

Of the seven children with mosaic *DICER1* hotspot mutations, two had both type II and type Ir PPB foci and one of these children ultimately succumbed to metastatic disease. The remaining five presented with only cystic PPB (type I or Ir) rather than sarcomatous disease (type II or type III) and those five have survived to date. This does not necessarily reflect a tendency to lung disease with less malignant potential. In general, the hotspot mosaic children were diagnosed very early because of unusually numerous, bilateral lung cysts that caused obvious breathing difficulty. All were treated promptly and closely monitored from that time forward. In contrast, children with a single focus of type I PPB, as frequently seen with germline LOF mutations, may go undiagnosed and continue to progress for many months, resulting in higher incidences of sarcomatous disease and metastasis, greater resistance to treatment and lower survivals. Though six of the seven hotspot mosaic children are alive, their clinical experiences have been complicated and arduous because of extensive lung resections and additional disease foci in organs besides lung ( Figure 3). Each has undergone multiple major surgeries and chemotherapies.

### Tumor-specific, biallelic *DICER1* mutations give rise to sporadic (non-syndromic) PPB

In twelve children, we identified biallelic *DICER1* mutations present at high allele frequencies in a PPB tumor, but not detectable in blood even with the benefit of high-depth NGS ( Table S9). Tumors from these children had an RNase IIIb hotspot missense mutation and either a nonsense LOF mutation (n = 5) or allele loss (n = 7). All twelve children presented with a single PPB tumor and none developed additional foci of disease in the lungs or other organs over the course of subsequent follow-up. None had family members with any form of DICER1 syndromic disease. This is consistent with occurrence of both an RNase IIIb hotspot mutation and a LOF mutation or allele loss within a single, highly localized clone of somatic cells which then gave rise to the tumor. Tumors of this kind should be recognized as sporadic PPB, not indicating DICER1 syndrome. Absence of additional disease foci is a predictable outcome if both *DICER1* mutations are restricted to the initial site of tumorigenesis. However, the absence of additional disease foci among children in this category did not indicate less dangerous disease. Of the 12 patients, 11 had advanced PPB (type II or III), and two succumbed ( Table 1).

### Currently unresolved cases

Ten PPB probands in our cohort are negative for predisposing *DICER1* mutations detectable in blood DNA by Sanger sequencing or NGS of coding exons. All of these children had a single focus of disease, and thus may be sporadic cases involving tumor-specific, biallelic *DICER1* mutations, but tumor tissue is either not available or not of sufficient quality to confirm this by sequencing. Clinical features of the ten unresolved cases and the status of further analyses pending or completed, including tumor sequencing, NanoString copy number assay and germline sequencing for additional candidate loci, are summarized in Table S10.

Patient information dataset Excel file with deidentified raw data for patient ages at diagnosis and numbers of disease foci, and statistical analyses ^48^. Click here for additional data file. Copyright: © 2018 Brenneman M et al. 2018 Data associated with the article are available under the terms of the Creative Commons Zero "No rights reserved" data waiver (CC0 1.0 Public domain dedication).

## Discussion

### Genotype-phenotype correlation of predisposing mutations in PPB/DICER1 syndrome

All germline *DICER1* truncating mutations are predicted to be essentially equivalent in their effect: complete or near-complete loss of function in miRNA processing. This prediction is based partly on nonsense-mediated decay, but also reflects the functional domain structure of the *DICER1* protein. All truncating mutations so far identified in PPB/ *DICER1* syndrome patients interrupt the open reading frame before the end of the critical RNase IIIb domain ( Figure 1, Table S3). Neomorphic RNase IIIb domain function (skewed 5p/3p miRNA production) is a recurring feature of *DICER1* tumors, and it is plausible that loss of all wildtype RNase IIIb function is required for it to become tumorigenic in lung and other organ sites most frequently affected. Presumed equivalence of all truncating mutations is consistent with clinical findings: no correlations are apparent between locations of germline truncating mutations within the *DICER1* gene and clinical features such as age of onset, number of disease foci, specific tissue sites involved or survival. Non-truncating germline mutations are too rare for correlations with clinical presentations or outcomes to be ascertained.

The natural history of PPB indicates a multistep genetic pathogenesis, and so it is not surprising that in some cases where no germline *DICER1* mutation can be detected, one of the two different kinds of “hits” required for tumorigenesis in lung was acquired during embryogenesis in the form of somatic mosaicism. Mosaic mutations may ultimately prove important in the pathogenesis of many other sporadic childhood neoplasias, as demonstrated recently for retinoblastoma ( *RB1*) ^49^.

Mosaicism for RNase IIIb domain hotspot missense mutations defines a special category of *DICER1* syndrome patients that are phenotypically distinct from those who bear germline or mosaic LOF mutations. RNAse IIIb hotspot mutations have not been encountered as inherited alleles in this study or others, which suggests they are inviable ^4, 8, 10, 11, 14– 16, 21, 26– 30, 34, 46^. In addition to the seven mosaic RNase IIIb hotspot patients in our cohort, three apparently similar cases have been reported ( Table 2). Klein *et al.* described two infants with bilateral Wilms tumor and multiple cysts of the kidneys and lungs ^46^. Each child was found to be mosaic for a *DICER1* RNase IIIb domain missense mutation, although in one case the mutation was at D1713; also an acidic residue within the RNase IIIb catalytic cleft, but not a well-established hotspot (reported only once before, also in a Wilms tumor ^16^). De Kock *et al.* described an infant with pituitary blastoma and bilateral cysts of the kidneys and lungs in whom a *de-novo* hotspot mutation was detected at high allele frequency in blood as well as tumor ^11^.

Clinically, mosaic hotspot patients are distinguished by two features: *i.)* consistently early presentations of neoplastic disease, often by one year of age, and *ii.)* numerous discrete foci of disease developed concurrently or successively, usually involving more than one syndromic tissue/organ site ( Figure 3, Table 2). The two features are related and can be interpreted within the conceptual framework of the emerging model for *DICER1* syndrome pathogenesis, which provides important insight as to how tumor suppression by *DICER1* fails ^6, 26, 33, 35, 36^. *DICER1* is not a classical tumor suppressor gene for which “two hits” – loss of function in both alleles – are required to allow tumorigenesis. Neither is it haploinsufficient in the usual sense, *i.e.*, that cells with only one expressed allele make wild-type protein, but not in sufficient quantity to fulfill its function. Rather, it is neomorphic function by mutant *DICER1* protein, with substitutions of key amino acids in the RNase IIIb domain that causes tumor suppression to falter when it is not masked by expression of wild-type *DICER1* protein. Unmasking of an RNase IIIb hotspot mutation may arise through any form of LOF mutation in the wild type allele, including allele loss. The two mutational events, RNase IIIb missense and LOF, may occur in either order and both are generally required to foment the initiation of tumorigenesis in most organ sites. However, as outlined below, RNase IIIb hotspot mutation is a low-probability event and LOF mutation is, relatively, a very high-probability event. The projected consequence of these lopsided probabilities is that occurrence of an RNase IIIb hotspot mutation becomes the rate-limiting step in onset of pathogenesis.

### Rationale for the distinctive phenotype of mosaicism for RNase IIIb hotspot mutations

The RNase IIIb domain hotspots in *DICER1* are a diminutive mutational target; five codons within an open reading frame of 1922 codons (0.26%). Moreover, molecular mechanisms by which RNase IIIb hotspot missense mutations can arise are restricted to those errors of DNA replication and/or DNA repair that produce nucleotide substitution without disturbing the open reading frame. There are 36 possible single-nucleotide changes that can produce amino acid substitutions at these five codons, and only a subset of them has ever been identified in *DICER1* syndrome tumors. The spectrum of pathogenic RNase IIIb hotspot mutations is thus very narrow. In contrast, the spectrum of possible LOF mutations is broad and mechanistically diverse. Of the 1922 codons in the *DICER1* open reading frame, 675 can be converted to a stop codon by a single nucleotide change. A subset can be converted in more than one way, giving a total of 736 possible single nucleotide changes that result in a nonsense mutation. Among the other 16,562 possible single nucleotide changes in the *DICER1* open reading frame, presumably some would be missense mutations that disrupt *DICER1* protein function. The five non-hotspot missense mutations we detected as germline alleles in PPB probands are likely examples ( Figure 2). The individual nucleotides of the *DICER1* open reading frame present 5766 point locations at which insertion or deletion of one or a few nucleotides can shift reading frame. An additional 104 bases comprise canonical splice sites of the 26 *DICER1* introns, where small sequence changes may result in exon skipping, with or without frameshift. The possibilities for LOF mutations also include larger intra-locus deletions or inversions, translocations that interrupt the locus, and allele loss through copy-neutral loss of heterozygosity (which can arise by several mechanisms), segmental deletions or complete loss of chromosome 14. Absolute frequencies of these diverse *DICER1* mutational mechanisms in a particular cell lineage cannot be modeled precisely, but it becomes clear that the aggregate likelihood of all possible LOF mutations is vastly greater than the likelihood of a neomorphic mutation in one of the five hotspot codons.

It follows that in a developing embryo or child with a germline (or mosaic) *DICER1* LOF mutation, “second hits” occurring in a somatic cell will almost always be another LOF mutation, usually resulting in cell death or limited proliferation at most. Rarely, a second hit will be an RNase IIIb hotspot missense mutation, which allows for continuing cell viability and growth, though at the cost of skewed miRNA processing that may ultimately promote tumorigenesis in the surviving clones of cells. However, the low likelihood of incurring an RNase IIIb hotspot missense mutation in somatic cells means that months, years or a lifetime may elapse before one occurs. Further, the developmental context in which a second, hotspot mutation occurs may be important. There are apparently windows of risk for transformation, perhaps coinciding with certain periods of organ/tissue development when an onco-fetal gene program is normally active and subject to miRNA modulation, *i.e.*, lung, kidney and brain in the embryo; uterine cervix and ovaries in pubertal girls ^1– 3, 8, 9, 50^. A low probability of RNase IIIb hotspot mutations as second hits during windows of risk may underlie the low penetrance and variable expression of familial LOF mutations in *DICER1* syndrome.

For a developing child with a mosaic RNase IIIb hotspot mutation, the prospects are radically different. Somatic cells that bear the RNase IIIb hotspot mutation, masked by a wild type allele, will be viable and non-tumorigenic unless and until they sustain a second hit. However, cells with a preexisting RNase IIIb hotspot mutation are at high aggregate risk of acquiring a subsequent LOF mutation, because it can take any of the myriad forms outlined above. The probability of a secondary LOF mutation occurring during expansion of any given cell lineage over the course of prenatal and postnatal development is relatively high, and independent LOF mutations in multiple lineages may occur. If sufficient fractions of cells in critical lineages are affected, disturbed regulation of developmental gene expression programs arising from defective miRNA processing may be lethal *in utero*. For surviving children, onsets of tumorigenesis will tend to be early and, depending on embryonic distribution of the RNase IIIb hotspot mutation, foci of tumorigenesis may arise in one or more organ sites characteristic of *DICER1* syndrome. Additionally, we hypothesize that in mosaic hotspot children, wider tissue/organ distribution of aberrant miRNA processing during development may produce syndromic features occuring vary rarely or not at all in children with predisposing LOF mutations, such as juvenile-type small intestinal polyps, or the generalized somatic overgrowth noted in two cases by Klein *et al* ^46^.

The general trend that both RNase IIIb hotspot and LOF/allele loss mutations in *DICER1* are required to promote tumorigenesis has some evident exceptions among certain tumors less commonly associated with *DICER1* mutation. The Foulkes lab has described a series of pineoblastomas in which RNase IIIb hotspot mutations are clearly absent and *DICER1* function seems to have been lost completely, through either a truncating mutation in conjunction with allele loss or two successive truncating mutations ^21^. This implies that in the pineal gland specifically, the role of *DICER1* more closely resembles a classical tumor suppressor, such that complete loss of function enables tumorigenesis ^21^. Conversely, there are reports of tumors in which a *DICER1* hotspot missense mutation was confirmed, but no LOF mutations could be identified through sequencing of the coding exons and allele loss was not confirmed. Several independently reported cases of Wilms tumors fall into this category ^46, 51, 52^, as well as two non-epithelial ovarian tumors (one primitive germ cell tumor of yolk-sac type and one juvenile-type granulosa cell tumor ^26^). It is difficult to rule out the presence of cryptic LOF mutations involving change in non-coding regulatory elements or structural rearrangements of the *DICER1* locus not detectable by exon sequencing, and this remains a possibility in these cases. However, an alternative hypothesis must also be entertained: that for some tissue/organ sites, at some times during development, a *DICER1* hotspot missense mutation can be sufficient to promote tumorigenesis even in the presence of an expressed wild-type allele ^46^, ^52^. This might occur if DICER1 protein with amino acid substitutions at critical sites in the RNase IIIb domain can exert a dominant-negative effect over wild-type DICER1 in miRNA processing. For the closely related miRNA-processing protein DROSHA, Rakheja *et al.* presented compelling evidence that substitution at E1147, an analogous metal-binding residue in the conserved RNase IIIb catalytic cleft, dominantly suppresses the function of wild-type DROSHA in processing primary miRNA transcripts ^52^.

### Implications for mutation testing, clinical evaluation, and genetic counseling

Recent publications have outlined general recommendations for mutation detection and clinical evaluation for syndromic disease in patients with suspected *DICER1* syndrome and family members ^9, 51– 55^. Here we add considerations of risk for multifocal disease and reproductive transmission of *DICER1* mutations based on mutation category.

Most predisposing *DICER1* mutations are germline and detectable by targeted Sanger sequencing from blood. Initial testing should include parents, to distinguish inherited from *de novo* mutations. Sanger sequencing will usually suffice to detect a parental mutation that is also constitutional, but may fail to detect mosaicism. There is growing appreciation that apparently *de novo* mutations in children with genetic disease sometimes stem from mosaicism in a parent, which can often be detected by more sensitive methods ^56^. For eight patients with apparently *de novo* mutations in this cohort, we found no conclusive evidence of mosaicism in parents by resequencing with high-depth NGS, but this limited finding does not exclude the possibility of parental mosaicism for families evaluated in the future.

For those patients who have a tumor with confirmed *DICER1* mutation(s), but test negative for germline mutation by Sanger sequencing from blood, it will be important to distinguish as rigorously as possible between tumor-specific, biallelic mutations (sporadic PPB) and the presence of underlying mosaicism. Mutations confined to the tumor will confer no risk for new foci of primary disease in the proband, and family members including potential offspring will be unaffected. Mosaicism, whether for an LOF mutation or an RNase IIIb hotspot mutation, will confer some degree of risk for additional syndromic neoplasias. The very rare child who presents in early infancy with multiple *DICER1* syndromic neoplasias should raise suspicion of a mosaic RNase IIIb hotspot mutation. It may be impossible to unequivocally rule out mosaicism, but techniques such as targeted resequencing by high depth NGS in blood plus other available non-tumor specimens ( *e.g*., buccal cells or normal adjacent tissue recovered at tumor resection) can greatly improve diagnostic confidence, particularly with respect to RNase IIIb hotspot mutations. For patients who have more than one focus of disease but no germline or mosaic LOF mutation identifiable by targeted NGS of exons, testing for intragenic deletions or larger genomic alterations is recommended.

Patients carrying mosaic RNase IIIb hotspot mutations are predicted, on the basis of both clinical observations and mechanistic rationale, to have extraordinarily high risk as a group for developing multiple disease foci; approaching 100%. It will not be possible to predict individual risk for multifocal disease by allele frequency in blood or any other single specimen of normal cells, as this will not reveal the extent to which other somatic lineages harbor the mutation. Mosaic RNase IIIb hotspot patients will benefit from the most proactive program of family education and surveillance. The International PPB Registry recommends that potential benefits of renal ultrasound and surveillance chest CT be discussed with the family ^54, 55^. The frequency of follow-up chest CTs and chest radiographs should be determined individually, based on patient age, medical history and previous imaging results. Continuing evaluations should include a yearly complete review of systems by a clinician familiar with *DICER1* syndrome; yearly screening for ovarian SLCT with review of systems for endocrine dysfunction and pelvic ultrasound for females from early childhood through adulthood; yearly ophthalmologic examination and yearly thyroid examination by palpation and/or ultrasound. Pituitary blastoma and pineoblastoma are rare even in *DICER1* syndrome and typically limited to the infant and young child. There is no consensus at this time on screening for intracranial neoplasms.

As prospective parents, patients who are mosaic for a *DICER1* mutation face a theoretical risk for transmitting the mutation of up to 50%, depending upon whether and at what frequency it is present in germ cells. For carriers of a mosaic LOF mutation, the consequences of transmission will be similar to those of a germline LOF mutation carrier. For carriers of a mosaic RNase IIIb hotspot mutation, it is uncertain whether transmission could result in a live birth. The absence of RNase IIIb hotspot mutations as inherited alleles in all published studies implies they preclude development to term, but this remains speculative. The mosaic hotspot mutation identified in patient 101 of this cohort was discernable in blood by Sanger sequencing and present at 15% of NGS read counts in normal lymph node tissue ( Table 2). Similarly in the two Wilms tumor patients reported by Klein *et al.* and one pituitary blastoma patient described by De Kock *et al.*, *de-novo* hotspot mutations were readily detected in blood by Sanger sequencing ^11, 46^. Whether the latter case is truly germline or mosaic with high representation in the blood lineage was unclear. Nonetheless, it is clear from these examples that human embryogenesis can tolerate a *DICER1* hotspot mutation at high allele frequency in at least some cell lineages. It thus seems possible, though unlikely, that an inherited RNAse IIIb hotspot mutation could be viable.

## Data availability

The data referenced by this article are under copyright with the following copyright statement: Copyright: © 2018 Brenneman M et al.

Data associated with the article are available under the terms of the Creative Commons Zero "No rights reserved" data waiver (CC0 1.0 Public domain dedication). 

The ClinVar accession number(s) for the variant sequences reported in this paper are SCV000195560-SCV000195643.

*F1000Research*: Dataset 1. Patient information dataset, 10.5256/f1000research.6746.d80768 ^48^

## Web resources

ClinVar database, http://www.ncbi.nlm.nih.gov/clinvar/

Online Mendelian Inheritance in Man (OMIM), http://www.omim.org/

PPB Genetic Study In: Clinical Trials.Gov available from, http://clinicaltrials.gov/show/NCT00565903

International PPB Registry, http://www.ppbregistry.org

NCI *DICER1* Phenotype Study, http://dceg.cancer.gov/research/clinical-studies/ *DICER1*-ppb-study

## Supplemental data

**Supplemental data for ‘Temporal order of RNase IIIb and loss-of-function mutations during development determines phenotype in pleuropulmonary blastoma/ *DICER1* syndrome: a unique variant of the two-hit tumor suppression model’.**

Supplemental data file comprises ten tables:

Table S1. *DICER1* coding region amplicons for Ion Torrent sequencing

Table S2. *DICER1* probes for NanoString copy number assays

Table S3. Summary of germline *DICER1* loss-of-function mutations identified in PPB children

Table S4. Non-truncating germline *DICER1* mutations – additional details

Table S5. High-depth sequencing in parents of children with presumed *de novo DICER1* germline mutations

Table S6. Clinical features of children with germline *DICER1* loss of function mutations

Table S7. Summary of somatic *DICER1* RNase IIIb domain “hotspot” mutations identified

Table S8. Sequence results from children with mosaic *DICER1* loss of function mutation

Table S9. Sequence results from children with tumor specific, biallelic *DICER1* mutations

Table S10. Clinical features of 10 unresolved cases; PPB children who tested negative for germline *DICER1* mutation
