# Supplementary material for: Temporal order of RNase IIIb and loss-of-function mutations during development determines phenotype in pleuropulmonary blastoma / DICER1 syndrome: a unique variant of the two-hit tumor suppression model
Source: F1000Res. 2018 Jan 12;4:214. Originally published 2015 Jul 10. [Version 2] doi: 10.12688/f1000research.6746.2 (PMC4712775; doi:10.12688/f1000research.6746.2)
Supplement: Supplementary file 2 [file f1000research-4-14447-s0001.tgz › 6b7e04a3-b9b0-4eb6-ada7-498ed862d842.docx]

**Table S1. *DICER1* coding region amplicons for Ion Torrent sequencing**

| Amplicon ID | Genomic coordinates |
| --- | --- |
| DICER1 AMPL258536787 | chr14:95556744-95556907 |
| DICER1 AMPL258543593 | chr14:95556901-95557048 |
| DICER1 AMPL258562985 | chr14:95557286-95557402 |
| DICER1 AMPL258563935 | chr14:95557397-95557573 |
| DICER1 AMPL258543118 | chr14:95557567-95557739 |
| DICER1 AMPL258471776 | chr14:95560195-95560352 |
| DICER1 AMPL258494006 | chr14:95560346-95560522 |
| DICER1 AMPL258467746 | chr14:95562134-95562304 |
| DICER1 AMPL258492004 | chr14:95562300-95562475 |
| DICER1 AMPL258506470 | chr14:95562469-95562631 |
| DICER1 AMPL258478107 | chr14:95562625-95562741 |
| DICER1 AMPL258491410 | chr14:95562737-95562903 |
| DICER1 AMPL258504089 | chr14:95562899-95563033 |
| DICER1 AMPL258505507 | chr14:95563029-95563108 |
| DICER1 AMPL258453538 | chr14:95566073-95566155 |
| DICER1 AMPL258461486 | chr14:95566149-95566298 |
| DICER1 AMPL258571359 | chr14:95569580-95569736 |
| DICER1 AMPL258582162 | chr14:95569731-95569893 |
| DICER1 AMPL258590138 | chr14:95569887-95570013 |
| DICER1 AMPL258595555 | chr14:95570007-95570158 |
| DICER1 AMPL258572769 | chr14:95570153-95570324 |
| DICER1 AMPL258579260 | chr14:95570318-95570488 |
| DICER1 AMPL258556908 | chr14:95571281-95571417 |
| DICER1 AMPL258561328 | chr14:95571411-95571608 |
| DICER1 AMPL258453467 | chr14:95572002-95572083 |
| DICER1 AMPL258457579 | chr14:95572073-95572159 |
| DICER1 AMPL258456281 | chr14:95572349-95572491 |
| DICER1 AMPL-E1801 | chr14:95572485-95572582 |
| DICER1 AMPL258551772 | chr14:95573904-95574068 |
| DICER1 AMPL258553866 | chr14:95574062-95574214 |
| DICER1 AMPL258453534 | chr14:95574184-95574266 |
| DICER1 AMPL258457777 | chr14:95574260-95574395 |
| DICER1 AMPL258466438 | chr14:95574391-95574455 |
| DICER1 AMPL258576459 | chr14:95574634-95574713 |
| DICER1 AMPL258581441 | chr14:95574707-95574865 |
| DICER1 AMPL258453678 | chr14:95577591-95577693 |
| DICER1 AMPL258471633 | chr14:95577689-95577860 |
| DICER1 AMPL258554018 | chr14:95578401-95578525 |
| DICER1 AMPL258554738 | chr14:95578521-95578614 |
| DICER1 AMPL258566737 | chr14:95579374-95579527 |
| DICER1 AMPL258568190 | chr14:95579523-95579693 |
| DICER1 AMPL258504471 | chr14:95581935-95582074 |
| DICER1 AMPL258510835 | chr14:95582070-95582213 |
| DICER1 AMPL258453707 | chr14:95582769-95582904 |
| DICER1 AMPL258468517 | chr14:95582898-95583045 |
| DICER1 AMPL258457405 | chr14:95583908-95583990 |
| DICER1 AMPL258467641 | chr14:95583984-95584153 |
| DICER1 AMPL258459541 | chr14:95590482-95590596 |
| DICER1 AMPL258479597 | chr14:95590590-95590750 |
| DICER1 AMPL258491239 | chr14:95590744-95590916 |
| DICER1 AMPL258500149 | chr14:95590910-95591048 |
| DICER1 AMPL258453466 | chr14:95592905-95593015 |
| DICER1 AMPL258453657 | chr14:95593015-95593095 |
| DICER1 AMPL258556387 | chr14:95595742-95595912 |
| DICER1 AMPL258560606 | chr14:95595906-95596020 |
| DICER1 AMPL258453563 | chr14:95596363-95596434 |
| DICER1 AMPL258454096 | chr14:95596434-95596554 |
| DICER1 AMPL258453557 | chr14:95597804-95597932 |
| DICER1 AMPL258462374 | chr14:95597928-95598012 |
| DICER1 AMPL258454257 | chr14:95598825-95598946 |
| DICER1 AMPL258466632 | chr14:95598942-95599035 |
| DICER1 AMPL258453778 | chr14:95599644-95599742 |
| DICER1 AMPL258459568 | chr14:95599736-95599822 |

**Table S2. *DICER1* probes for NanoString copy number assays**

| Probe ID | Genomic Coordinates | Target Sequence | Tm CP | Tm RP |
| --- | --- | --- | --- | --- |
| DICER1_166.1:265 | chr14:95552495-95552594 | GATCATTTCTGCTACCACTATTTAAACGTTATTACCCACTTGAATTGAAAAAAGATGAAAGGACACAAAGTGAACAGACGATAACTTTATTGGAGATTTA | 80 | 80 |
| DICER1_4798.1:60 | chr14:95556922-95557012 | ACCAACACCTTTAAATTTCCCCTTTCCTACTACTTCCACAGTGACTCTGACCTTCCCGTCGTAAGTTCTCTCAGCCGGGCTGTAAAAAATCCAAACAGCT | 84 | 85 |
| DICER1_5038.1:401 | chr14:95557503-95557592 | TGTGGGTTTTTTTCTTTCTAAAGGGAGCCAACAATACCTATTAGTGGCCGCATCATGGGATAGTACACCTGCCAGACTGTCTCCAGTGACATCCCACTAT | 85 | 85 |
| DICER1_7826.1:270 | chr14:95560160-95560259 | ACTCTGAAACTACAGAGACTCCTAGTTAGACCACTTTTTTCAACATCGTTTTGAACAGCACTAACCTCAGAATCCATTCCTTGCATTTCATTCTTCTCAA | 82 | 83 |
| DICER1_10332.1:18 | chr14:95562425-95562509 | TTTGTTGGCTGTTGAAATTCTCCCGAGTAGGGCACAGGGCCTTTTCCCGATCAGTCCTTTTAATTACCGGGAGCACCTTCAGCCCCAGTGAACAGAGGAA | 87 | 87 |
| DICER1_13708.1:375 | chr14:95566147-95566236 | GCTTTTGTCTTGATTTACTACATAACCAGGAGGAAGCCAATTCACAGGGGGATCAAATATTGACACCACCATGCGGCTGGGTAGTCCCTTCTTTTTTCCA | 85 | 85 |
| DICER1_17489.1:205 | chr14:95569763-95569857 | ATGCTTTAAAAAGGAGTCGCCAAGCATTTCAAGCCGCTCCAGGTTAAATCCATCACTAGCGTTTGACAGAGTCAAAGCCTGAAGAATAAGTCCAGGATTG | 85 | 85 |
| DICER1_18845.1:445 | chr14:95571364-95571443 | TTGCAAACAGGATCTCATGATCTGTGTTCTTTCTGGCTGACTGCACAGGCATACCTAAAATCCGCAGGAAGTGATCTGACTCCCACGCCAGCATCGCTGG | 85 | 85 |
| DICER1_19460.1:563 | chr14:95572087-95572186 | CTGATTCAAATGTCGAGGTGTCAAAAGATTAAGTCTGTAAGAATTCCAAAACAATTTTATCAAACACACAAAAATGAGTACATATTCACAGTGGTTCTCC | 81 | 80 |
| DICER1_20138.1:198 | chr14:95572400-95572499 | CCAGCAGTGGCTGGTTGAGATTGGTTAGGTCAAGGTTGTACTTTGTTTTATAATATTCTGCAAAAGTTTCATACTCAGGGGAAGGAAATTTACTGAGTGG | 86 | 81 |
| DICER1_21617.1:252 | chr14:95573933-95574032 | TTAAATACCTACCTTGGAATGATAACGGCATCTTGGTAATCTTCTAATTTAAAAACAAAGGGTGTTTCTTTTGTATACTTTGTACTGGGAATGCCTATGC | 80 | 82 |
| DICER1_22097.1:100 | chr14:95574261-95574360 | GGTTTAAATTCTAGTGCAGGTTTTTCAAGCCGAAGAATATGTGAGAATATATACTGGTGAAGTCTTGTAATCAACTCAAGCATTTGTAGAGACAACATGA | 81 | 82 |
| DICER1_22658.1:0 | chr14:95574722-95574821 | AGCTTCCGCCTTCTAAAGTTGAGTTCATCAGGTAAAGGTGTAGTTAAAACCATTCCTATCACATACAGGTAACAGGGCTGATCAGGTCTGGGATAACTAT | 83 | 84 |
| DICER1_25461.1:103 | chr14:95577628-95577717 | TTTACTCTGTTCTAACCAATACTAACTGCTTTTGGGTAGCACTGCCTTCGTTTCGTGGAACCTGGTCTTCCTGGAACACTGGTCTCTTCTTCATCATGCA | 84 | 85 |
| DICER1_26505.1:0 | chr14:95578569-95578667 | AGCTCATTGGTGGACCCTGAAAATAACAAAAACCTTTCCATTATATATGCACATCGCTGTATTACCTCTAGCACATGAAACACCTATGAAACCACTGCCC | 82 | 85 |
| DICER1_27301.1:29 | chr14:95579394-95579493 | TAAAAGATAACAATCATTTCTTCTTCTAAACTTACAACAATGGAGGCTCGAAGAGGTGAGTTAATTGGCAGATAAAGAGTTGAATAAAATGTACCATCAG | 80 | 80 |
| DICER1_29526.1:460 | chr14:95582055-95582144 | CATCGTCAGGCCTCAACACATATGGTGGGAAAACGTCATCATCATCCATGACAGGATCAATGTCAGTCTCACCAGTATCAACCGACTTGGAACACTTGTT | 85 | 85 |
| DICER1_30735.1:121 | chr14:95582923-95583019 | GTGGGCAAATCAAAACGAACCACCAAGTTGCATTTTGGTATATCAACACCCTCTTCTACAATACTTGTTGCAATAAGCAGGTTGGTCTCATGTGCTCGAA | 84 | 84 |
| DICER1_31749.1:146 | chr14:95583965-95584058 | CTCTTCCTGTTTTCTGAATTCTGCTTCCATCTGTTTGTTGCGAGGCTGATTCTTCCCAATGCCATGTCCAGTTATGAAATTGCTACTGATATAAGCCAGC | 84 | 85 |
| DICER1_38392.1:270 | chr14:95590726-95590825 | ACTGCTGTCGCTCATATGGTTTATATTTGCGTAAGATTTCGAGCAGTTTGATTACTTTAGGAGTTACAAATTTCAGGTCAAGTGAGGCAGGTGAGAAGTG | 84 | 83 |
| DICER1_40809.1:115 | chr14:95592988-95593087 | GTGCTTCTTCTAATTCCATCAGCAGTCTTTCATAAAGCCCACTTCTGTCAGTAAATGGTCCACAATCCACCACAATCTCACATGGCTGAGAAGTATACCT | 84 | 85 |
| DICER1_43720.1:66 | chr14:95595850-95595949 | CTTAAGAATTTTCTCTAGTTTCTGAATCTTTTCTTCCAATTCCTCTGGATCACATTTCCCATTTAAAATGGAAGCAGTTAGTCCCAAAATGCGAGGACAT | 79 | 84 |
| DICER1_43904.1:988 | chr14:95596956-95597045 | GTGCTTTATTAATGAGGACTTATCCCAGTATGTGAAGCACACTAGTAGGCTGGGCTGTTTCAGTTCAGTGAGGAAGCGCCTCTTAATCACTGTGTATCCC | 83 | 85 |
| DICER1_45649.1:9 | chr14:95597722-95597821 | GCTGATTAAGTATAGGAAATTAGTTTTCAAACCTAAATCAGACAACCAAGGCTACAGATCATCTTACAAACCAAGTCAAGAACTTGTAGGGATTTATAAA | 79 | 80 |
| DICER1_46492.1:234 | chr14:95598790-95598889 | GTTTAAACATAAAATCCCATCCAATTTCCCCTGCACAACTTGATAAAATAATTTTTTATTACCAGAGTTGACCAAGAACACCGTCCTTTTTCCATTTCTG | 81 | 82 |
| DICER1_47309.1:214 | chr14:95599587-95599686 | CAACTATATGCTAATGTATTCCATTTTAGTGTAGAATGCTCCAGTATTAGTGTTCGCATTAGTACCTGATATTTTCTTGGCGTATAAATGTTATCATGAA | 78 | 80 |
| DICER1_50874.1:140 | chr14:95603083-95603172 | TAGTCCCATTTGCTGCTGCAATTACACTACACAGACCCATGAAAAGATCCAGCCTTAGGATGAAACCAGCACCGCAGATGGCAAAAATAAAGAACCTGGG | 84 | 85 |
| DICER1_55023.1:0 | chr14:95607087-95607186 | AGCTTCTGCTCTAAAAATAAATGCAAAAATAGCCAAGTTTAAGATTCCTGTAAACTGAACAGAAACACATCTACAAGAGAGCCCTACTGATATGAAGAGT | 80 | 82 |
| DICER1_55787.1:168 | chr14:95608019-95608118 | CCTCAAAATGCTGATTCTAAGTTCTTTCAGAGTTTCCAAGTCTGTATTAATTCATACCCTAGGTAACCTAAAAGCAATCAGAGAGAAATTTTAGAAGAGA | 80 | 80 |
| DICER1_70802.1:63 | chr14:95622941-95623018 | AAATCAAAAACCCTGAAGAAAGGGTCCTCCGCAGGGTCTAGGCATTTTGGCACAAAAACATCTGAGGGGAGAGGCAAAAGAATGGCCTCGGCCCCTGCCC | 86 | 85 |

Abbreviations: Tm CP, melting temperature of capture probe; Tm RP, melting temperature of reporter probe.

**Table S3. Summary of *DICER1* loss-of-function mutations identified**

| *DICER1* exon | Nucleotide change  (NM_177438.2) | Protein change^a^ (NP_803187.1) | Mutation type | Study ID |
| --- | --- | --- | --- | --- |
| 5 | c.559C>T | p.R187* | nonsense | 1 |
| 6 | c.629T>G | p.L210* | nonsense | 80 |
| intron 6 | c.735-1_741delGGTATACTinsA | exon 7 skip; fs* | 3' splice site | 50 |
| 7 | c.745C>T | p.Gln249* | nonsense | 5 |
| 7 | c.878_881delGAGA | p.R293Ifs*4 | frameshift | 37 |
| 8 | c.1144G>T | p.E382* | nonsense | 34 |
| 8 | c.1165_1174dupCCATATGAGC | p.R392Pfs3* | frameshift | 89 |
| 8 | c.1185_1187delTGAinsC | p.E396Kfs*3 | frameshift | 110 |
| 8 | c.1202dupA | p.Y401* | frameshift | 3 |
| 8 | c.1202dupA | p.Y401* | frameshift | 8 |
| intron 8 | c.1376+1G>T | exon 8 skip; fs* | 5' splice site | 25 |
| 9 | c.1408G>T | p.E470* | nonsense | 49 |
| 9 | c.1507G>T | p.E503* | nonsense | 11 |
| 10 | c.1525C>T | p.R509* | nonsense | 4 |
| 10 | c.1525C>T | p.R509* | nonsense | 65 |
| 10 | c.1630C>T | p.R544* | nonsense | 48 |
| 10 | c.1651G>T | p.G551* | nonsense | 33 |
| 10 | c.1684_1685delAT | p.M562Vfs*11 | frameshift | 21 |
| 10 | c.1694_1695delAT | p.T566Rfs*7 | frameshift | 54 |
| 10 | c.1716delT | p.F572Lfs*14 | frameshift | 93 |
| 10 | c.1745T>C | p.I582T | missense | 36 |
| intron 10 | c.1752+1delG | exon 10 skip | 5' splice site | 108 |
| 11 | c.1787delC | p.T596Mfs*21 | frameshift | 67 |
| 11 | c.1827_1828insAG | p.V610Rfs*8 | frameshift | 86 |
| 11 | c.1870C>T | p.R624* | nonsense | 66 |
| 11 | c.1870C>T | p.R624* | nonsense | 68 |
| 11 | c.1880_1883delTCAA | p.I627Tfs*22 | frameshift | 7 |
| intron 11 | c.1907+1delG | exon 11 skip; fs* | 5' splice site | 77 |
| 12 | c.1910dupA | p.Y637* | frameshift | 20 |
| 12 | c.1966C>T | p.R656* | nonsense | 35 |
| 12 | c.1966C>T | p.R656* | nonsense | 42 |
| intron 12 | c.2040+1G>T | exon 12 skip; fs* | 5' splice site | 13 |
| 13 | c.2062C>T | p.R688* | nonsense | 59 |
| 13 | c.2062C>T | p.R688* | nonsense | 81 |
| 13 | c.2062C>T | p.R688* | nonsense | 85 |
| 14 | c.2158_2159delGT | p.V720* | frameshift | 72 |
| 14 | c.2236A>G | p.R746G | missense | 92 |
| 14 | c.2243_2244delGCinsAA | p.C748* | frameshift | 45 |
| 14 | c.2243_2243insACTA | p.Y749* | frameshift | 38 |
| 14 | c.2247C>A | p.Y749* | nonsense | 6 |
| 15 | c.2392dupA | p.T798Nfs*33 | frameshift | 2 |
| intron 16 | c.2650+1G>T | exon 16 skip; fs* | 5' splice site | 73 |
| 17 | c.2749G>T | p.E917* | nonsense | 78 |
| 18 | c.2830C>T | p.R944* | nonsense | 16 |
| 18 | c.2830C>T | p.R944* | nonsense | 53 |
| 18 | c.2830C>T | p.R944* | nonsense | 60 |
| 18 | c.2830C>T | p.R944* | nonsense | 79 |
| 18 | c.2863delA | p.T955Pfs*26 | frameshift | 10 |
| 18 | c.2867_2869delCACinsAA | p.P956Qfs*25 | frameshift | 12 |
| 18 | c.2888_2889delCT | p.P963Rfs*3 | frameshift | 62 |
| 21 | c.3135_3137delATCinsTGAACTCATG | p.S1046Efs*47 | frameshift | 69 |
| 21 | c.3175dupT | p.Y1059Lfs*32 | frameshift | 9 |
| 21 | c.3273C>G | p.Y1091* | nonsense | 47 |
| 21 | c.3277_3280delAACT | p.N1093* | frameshift | 71 |
| 21 | c.3300delA | p.K1100Nfs*44 | frameshift | 41 |
| 21 | c.3515_3525 delTTACAGCAATTinsA | p.L1172Qfs*17 | frameshift | 30 |
| 21 | c.3534_3535insAA | p.S1179Nfs*14 | frameshift | 109 |
| 21 | c.3538_3539delTA | p.Y1180Qfs*10 | frameshift | 64 |
| 21 | c.3540C>A | p.Y1180* | nonsense | 43 |
| 21 | c.3540C>A | p.Y1180* | nonsense | 70 |
| 21 | c.3579_3580delCA | p.N1193Kfs*41 | frameshift | 23 |
| 21 | c.3589delT | p.C1197Afs*6 | frameshift | 24 |
| 21 | c.3658C>T | p.Q1220* | nonsense | 58 |
| 21 | c.3676G>T | p.E1226* | nonsense | 39 |
| 21 | c.3726C>A | p.Y1242* | nonsense | 82 |
| 21 | c.3777dupC | p.V1260Rfs*19 | frameshift | 15 |
| 21 | c.3777dupC | p.V1260Rfs*19 | frameshift | 29 |
| 21 | c.3875_3878delCCAA | p.P1292Lfs*9 | frameshift | 76 |
| 22 | c.4190G>A | p.W1397* | nonsense | 63 |
| 23 | c.4309_4312delGACT | p.D1437Mfs*16 | frameshift | 84 |
| 23 | c.4309_4312delGACT | p.D1437Mfs*16 | frameshift | 87 |
| 23 | c.4407_4408delTT | p.P1471Ffs*5 | frameshift | 55 |
| 23 | c.4407_4410delTTCT | p.S1470Lfs*19 | frameshift | 17 |
| 23 | c.4407_4410delTTCT | p.S1470Lfs*19 | frameshift | 61 |
| 23 | c.4407_4410delTTCT | p.S1470Lfs*19 | frameshift | 75 |
| 23 | c.4426_4427insT | p.D1476Vfs5* | frameshift | 90 |
| 23 | c.4511delG | p.S1504Tfs*56 | frameshift | 83 |
| 23 | c.4517G>A | p.W1506* | nonsense | 52 |
| 23 | c.4605_4606delTG | p.C1535Wfs*3 | frameshift | 19 |
| 23 | c.4621A>T | p.K1541* | nonsense | 51 |
| 23 | c.4748T>G | p.L1583R | missense | 26 |
| 23 | c.4754C>G | p.S1585* | nonsense | 46 |
| 23 | c.4960_4961dupGA | p.D1654Efs*7 | frameshift | 22 |
| 23 | c.4960_4961dupGA | p.D1654Efs*7 | frameshift | 31 |
| 23 | c.5053C>T | p.Q1685* | nonsense | 91 |
| intron 23 | c.5095+1G>C | exon 23 skip; fs* | 5' splice site | 14 |
| 24 | c.5096-498_5364+356del | p.D1699Afs*1 | 1kb deletion | 88 |
| 24 | c.5104C>T | p.Q1702* | nonsense | 56 |
| 24 | c.5123G>A | p.G1708E | missense | 27 |
| 24 | c.5194dupC | p.L1732Pfs*33 | frameshift | 28 |
| 24 | c.5251_5255delAAGTA | p.K1751Rfs*12 | frameshift | 18 |
| 24 | c.5299delC | p.H1767Mfs*71 | frameshift | 74 |
| 24 | c.5315_5316delTT | p.F1772Cfs*6 | frameshift | 44 |
| 25 | c.5394delA | p.E1799Kfs*39 | frameshift | 32 |
| 25 | c.5465A>T | p.D1822V | missense | 40 |
| 25 | c.5465A>T | p.D1822V | missense | 57 |

a. Protein changes are inferred, *i.e.*, not supported by mRNA sequencing.

| Study ID | Exon | Nucleotide change (NM_177438.2) | Protein change^a^  (NP_803187.1) | DICER1  domain | PROVEAN score  (prediction) | SIFT  score  (prediction) | Evidence for  loss of function | Notes |
| --- | --- | --- | --- | --- | --- | --- | --- | --- |
| 36 | 10 | c.1745T>C | p.I582T | N-terminal helicase domain | -3.01 (deleterious) | 0.119  (tolerated) | No evidence beyond occurence in this PPB patient | May be a *de novo* mutation (no parental blood available). No syndromic disease reported in family members. |
| 108 | intron 10 5’ss | c.1752+1delG | Exon 10 skip; in-frame deletion of 81 amino acids | Between helicase and PAZ domains |  |  | Familial disease | Two family members have history of syndromic conditions, but mutation status unknown. |
| 26 | 23 | c.4748T>G | p.L1583R | RNase IIIa domain | -3.13  (deleterious) | 0.001  (damaging) | Segregates with familial disease (Hill *et al* ^4^) | Located at the C-proximal end of the RNase IIIa domain. |
| 27 | 24 | c.5123G>A | p.G1708E | RNase IIIb domain | -7.72  (deleterious) | 0.000  (damaging) | Present in *trans* to a  c.5438A>G; p.E1813G  RNase IIIb hotspot mutation in a PPB tumor (Pugh *et al* ^6^) | Pugh *et al* ^6^ proposed G1708E is a neomorphic allele, analogous to the highly recurrent hotspot mutation G1809R. However, it has not occurred in combination with any known LOF mutation in tumors reported to date. Its occurrence here in combination with a known hotspot mutation suggests G1708E may instead be an LOF mutation. |
| 40 | 25 | c.5465A>T | p.D1822V | RNase IIIb domain | -8.28  (deleterious) | 0.001  (damaging) | Segregates with familial disease.  Identified in two other probands (Slade *et al* ^14^) | Wild type aspartic acidic may participate in metal ion binding. Replaced with a non-polar valine. |
| 57 | 25 | c.5465A>T | p.D1822V | RNase IIIb domain | -8.28  (deleterious) | 0.001  (damaging) | Identified in two other probands (Slade *et al* ^14^) | Wild type aspartic acidic may participate in metal ion binding. Replaced with a non-polar valine. |

**Table S4. Non-truncating germline *DICER1* mutations – additional details**

Abbreviations: LOF, loss of function; PPB, pleuropulmonary blastoma

a. Protein changes inferred, *i.e.*, not supported by mRNA sequencing.

**Table S5. High-depth sequencing in parents of children with presumed *de novo DICER1* germline mutations**

| Proband Study ID | Proband mutation  (NM_177438.2) | Protein change^a^ | Mutation type | Frequency in  maternal blood  (variant / total reads) | Frequency in  paternal blood  (variant / total reads) |
| --- | --- | --- | --- | --- | --- |
| 10 | c.2863delA | p.T955Pfs*26 | frame shift | 0.131% (22/16785) | 0.064% (2/3117) |
| 13 | c.2040+1G>T | exon skip | frame shift | 0.003% (1/31802) | < 0.004% (0/29980) |
| 18 | c.5251_5255delAAGTA | p.K1751Rfs*12 | frame shift | < 0.011% (0/9339) | < 0.009% (0/11695) |
| 47 | c.3273C>G | p.Y1091* | nonsense | 0.004% (3/75810) | 0.010% (7/73141) |
| 50 | c.735-1_741delGGTATACTinsA | exon skip | frame shift | < 0.009% (0/12065) | < 0.001% (0/127538) |
| 52 | c.4517G>A | p.W1506* | nonsense | 0.043% (58/136311) | 0.028% (24/86854) |
| 59 | c.2062C>T | p.R688* | nonsense | 0.148% (3/2033)^b^ | 0.101% (1/988)^b^ |
| 69 | c.3135_3137delATCins10 | p.S1046Vfs*46 | frame shift | < 0.001% (0/160682) | < 0.004% (0/32378) |

a. Protein changes inferred, *i.e.*, not supported by mRNA sequencing.

b. variant frequencies above the estimated error rate for base substitutions (0.07%).

Note: With the exception of Study ID# 59, All variant sequence reads obtained in parental blood specimens occurred at frequencies below estimated error rates for the respective variant types in Ion Torrent sequencing, using the 200 bp *One Touch* kit: base substitutions, 0.07%; single-base deletions, 1.07%; single-base insertions, 1.76% (of bases in untrimmed reads).^54^

**Table S6. Clinical features of children with germline *DICER1* loss-of-function mutations**

| Study ID | Number of disease foci | PPB type | Other syndromic disease (in addition to PPB) | Syndromic condition in a family member | Survival status alive = 1 deceased = 0 | Parental screen:  positive  (inherited) = 1  negative  (de novo) = 0 |
| --- | --- | --- | --- | --- | --- | --- |
| 1 | 1 | II | N | N | 0 | 1 |
| 2 | 3 | II | lung cyst, thyroid nodule | Y | 1 | 1 |
| 3 | 2 | II | Thyroid follicular adenoma | N | 1 | 1 |
| 4 | 1 | III | N | NA | 0 | 1 |
| 6 | 2 | I | Thyroid follicular adenoma | Y | 1 | 1 |
| 7 | 1 | II | N | Y | 1 | 1 |
| 8 | 1 | II | N | N | 1 | 1 |
| 9 | 2 | I | lung cyst | N | 1 | NA |
| 10 | 3 | III | NCMH, lung cyst | N | 1 | 0 |
| 11 | 2 | I | lung cyst | Y | 1 | 1 |
| 12 | 3 | I | 2 lung cysts | N | 1 | 1 |
| 13 | 4 | III | NCMH, lung cyst, thyroid nodules | N | 1 | 0 |
| 14 | 2 | II | lung cyst | Y | 0 | 1 |
| 15 | 1 | II | N | Y | 1 | 1 |
| 16 | 2 | II | lung cyst | Y | 1 | 1 |
| 17 | 2 | III | NCMH | N | 1 | 1 |
| 18 | 1 | II | N | N | 0 | 0 |
| 19 | 1 | III | N | N | 1 | NA |
| 20 | 2 | I | ERMS, bladder | Y | 1 | 1 |
| 21 | 1 | III | N | N | 1 | 1 |
| 22 | 1 | II | (not known) | NA | 1 | NA |
| 23 | 4 | I | CBME, thyroid CA, lung cyst | N | 1 | 1 |
| 24 | 1 | III | N | N | 0 | 1 |
| 25 | 5 | II | NCMH, SLCT, peritoneal cyst, thyroid CA | Y | 1 | 1 |
| 26 | 1 | II | N | Y | 1 | 1 |
| 27 | 1 | III | N | NA | 1 | 1 |
| 28 | 2 | III | CN | Y | 1 | NA |
| 29 | 1 | II | N | Y | 1 | 1 |
| 30 | 2 | II | lung cyst | Y | 1 | 1 |
| 31 | 3 | III | lung cyst, CN | Y | 1 | 1 |
| 32 | 4 | II | 2 lung cysts, NCMH | N | 1 | 1 |
| 33 | 1 | III | N | NA | 0 | NA |
| 34 | 1 | I | N | NA | 1 | NA |
| 35 | 2 | II | ERMS, cervix | N | 1 | 1 |
| 36 | 3 | I | 2 lung cysts | N | 1 | NA |
| 37 | 1 | II | N | NA | 1 | 1 |
| 38 | 3 | I | 2 lung cysts | Y | 1 | 1 |
| 39 | 1 | III | N | Y | 1 | 1 |
| 40 | 1 | I | N | Y | 1 | 1 |
| 41 | 6 | I | CN, 4 lung cysts | Y | 1 | 1 |
| 42 | 3 | I | CN, lung cyst | Y | 1 | 1 |
| 43 | 1 | III | N | Y | 1 | 1 |
| 44 | 2 | I | lung cyst | Y | 1 | 1 |
| 45 | 2 | I | lung cyst | Y | 1 | 1 |
| 46 | 1 | III | N | N | 0 | 1 |
| 47 | 1 | III | N | N | 1 | 0 |
| 48 | 1 | III | N | Y | 1 | 1 |
| 49 | 1 | II | N | Y | 1 | 1 |
| 50 | 1 | Ir | N | N | 1 | 0 |
| 51 | 1 | II | N | N | 1 | 1 |
| 52 | 1 | III | N | N | 1 | 0 |
| 53 | 1 | I | N | Y | 1 | 1 |
| 54 | 1 | III | N | N | 0 | 0 |
| 55 | 3 | I | lung cyst, CN | Y | 1 | 1 |
| 56 | 3 | Ir | ERMS, SLCT | NA | 1 | 1 |
| 57 | 1 | II | N | NA | 1 | 1 |
| 58 | 2 | I | lung cyst | Y | 1 | 1 |
| 59 | 3 | III | 2 lung cysts | NA | 1 | 0 |
| 60 | 1 | III | N | Y | 0 | 1 |
| 61 | 1 | II | N | Y | 1 | 1 |
| 62 | 2 | III | ERMS | Y | 1 | 1 |
| 63 | 1 | II | N | NA | 1 | 0 |
| 64 | 1 | II | N | NA | 1 | 1 |
| 65 | 2 | Ir | CN | Y | 1 | 1 |
| 66 | 2 | II | CN | NA | 1 | 1 |
| 67 | 1 | II | N | NA | 1 | NA |
| 68 | 1 | III | N | Y | 1 | 1 |
| 69 | 3 | I | bilateral CN | N | 1 | 0 |
| 70 | 2 | Ir | CN | N | 1 | 1 |
| 71 | 2 | II | lung cyst | N | 1 | 1 |
| 72 | 2 | II | thyroid nodule | N | 1 | NA |
| 73 | 2 | I | lung cyst | Y | 1 | 1 |
| 74 | 1 | III | N | Y | 0 | 1 |
| 75 | 1 | II | N | Y | 1 | 1 |
| 76 | 2 | Ir | ERMS, cervix | N | 1 | 1 |
| 77 | 1 | I | N | Y | 1 | 1 |
| 78 | 1 | II | N | Y | 1 | 1 |
| 79 | 1 | II | N | NA | 1 | 1 |
| 80 | 2 | I | lung cyst | N | 1 | 1 |
| 81 | 1 | I | N | Y | 1 | 1 |
| 82 | 2 | II | thyroid CA | Y | 1 | 1 |
| 83 | 2 | I | lung cyst | NA | 1 | NA |
| 84 | 2 | Ir | CN | Y | 1 | 1 |
| 85 | 2 | I | pineoblastoma | NA | 1 | NA |
| 86 | 3 | Ir | CN, SLCT | NA | 1 | NA |
| 87 | 4 | I | ERMS (cervix), ERMS (bladder), thyroid Ca | N | 1 | 1 |
| 88 | 3 | Ir | 2 lung cysts | Y | 1 | NA |
| 108 | 3 | Ir | lung cyst, Wilms tumor | Y | 1 | NA |
| 109 | 1 | III | N | Y | 1 | 1 |
| 110 | 1 | III | N | Y | 1 | 1 |

Abbreviations: CA, carcinoma; CBME, ciliary body medulloepithelioma; CN, cystic nephroma; ERMS, embryonal rhabdomyosarcoma; N, none diagnosed to date; NCMH, nasal chondromesenchymal hamartoma; PPB, pleuropulmonary blastoma; SLCT, Sertoli-Leydig cell tumor

Note: This data set is limited, pursuant to concerns for potential identification of study participants based on particular combinations of clinical and pathologic features. Qualified investigators with specific questions about the study not answered by the data in these tables are invited to contact the International PPB Registry. The Registry will try to accommodate requests for additional data while preserving protected health information.

**Table S7. Summary of somatic *DICER1* RNase IIIb domain “hotspot” mutations identified**

| Hotspot codon | Nucleotide change (NM_177438.2) | Protein change (NP_803187.1) | Distribution | Tumor / tissue type(s) sequenced | Study ID |
| --- | --- | --- | --- | --- | --- |
| E1705 | c.5113G>A | p.E1705K | tumor-specific | PPB type II | 32 |
| (6 cases) | c.5113G>A | p.E1705K | tumor-specific | PPB type III | 33 |
|  | c.5113G>A | p.E1705K | tumor-specific | PPB type III | 46 |
|  | c.5113G>A | p.E1705K | tumor-specific | PPB type III | 97 |
|  | c.5113G>A | p.E1705K | mosaic | PPB type I, CN | 123 |
|  | c.5113G>A | p.E1705K | tumor-specific | PPB type II | 124 |
|  |  |  |  |  |  |
| D1709 | c.5125G>A | p.D1709N | tumor-specific | PPB type I | 5 |
| (11 cases) | c.5125G>A | p.D1709N | tumor-specific | PPB type II | 29 |
|  | c.5125G>A | p.D1709N | tumor-specific | PPB type II | 35 |
|  | c.5125G>A | p.D1709N | tumor-specific | PPB type II | 61 |
|  | c.5125G>A | p.D1709N | tumor-specific | PPB type II | 91 |
|  | c.5125G>A | p.D1709N | mosaic | Blood, metastatic PPB - brain | 102 |
|  | c.5125G>A | p.D1709N | mosaic | Blood, PPB type IR, CN, SIP | 103 |
|  | c.5125G>A | p.D1709N | tumor-specific | PPB type II | 111 |
|  | c.5125G>A | p.D1709N | tumor-specific | PPB type II | 112 |
|  | c.5125G>A | p.D1709N | tumor-specific | PPB type I | 121 |
|  | c.5126A>G | p.D1709G | mosaic | Blood, normal lymph node | 101 |
|  |  |  |  |  |  |
| G1809 | c.5425G>A | p.G1809R | tumor-specific | PPB type III | 13 |
| (15 cases) | c.5425G>A | p.G1809R | tumor-specific | PPB type III | 17 |
|  | c.5425G>A | p.G1809R | tumor-specific | PPB type III | 24 |
|  | c.5425G>A | p.G1809R | tumor-specific | PPB type III | 31 |
|  | c.5425G>A | p.G1809R | tumor-specific | PPB type II | 37 |
|  | c.5425G>A | p.G1809R | tumor-specific | PPB type III | 39 |
|  | c.5425G>A | p.G1809R | tumor-specific | PPB type III | 47 |
|  | c.5425G>A | p.G1809R | tumor-specific | PPB type III | 48 |
|  | c.5425G>A | p.G1809R | tumor-specific | PPB type II | 51 |
|  | c.5425G>A | p.G1809R | tumor-specific | PPB type III | 52 |
|  | c.5425G>A | p.G1809R | tumor-specific | PPB type III | 90 |
|  | c.5425G>A | p.G1809R | tumor-specific | PPB type III | 94 |
|  | c.5425G>A | p.G1809R | tumor-specific | PPB type III | 98 |
|  | c.5425G>A | p.G1809R | tumor-specific | PPB type II | 106 |
|  | c.5425G>A | p.G1809R | mosaic | PPB type II | 120 |
|  |  |  |  |  |  |
| D1810 | c.5428G>T | p.D1810Y | tumor-specific | PPB type III | 21 |
| (5 cases) | c.5428G>T | p.D1810Y | tumor-specific | PPB type II | 25 |
|  | c.5428G>T | p.D1810Y | tumor-specific | PPB type I | 95 |
|  | c.5428G>T | p.D1810Y | tumor-specific | PPB type III | 100 |
|  | c.5428G>T | p.D1810Y | mosaic | Blood, normal fallopian tube,  PPB type IR, CN, SLCT (two) | 104 |
|  |  |  |  |  |  |
| E1813 | c.5437G>C | p.E1813Q | mosaic | Blood, NCMH, SLCT (two), TCa | 105 |
| (7 cases) | c.5438A>G | p.E1813G | tumor-specific | PPB type I | 1 |
|  | c.5438A>G | p.E1813G | tumor-specific | PPB type III | 27 |
|  | c.5438A>G | p.E1813G | tumor-specific | PPB type III | 96 |
|  | c.5438A>G | p.E1813G | tumor-specific | PPB type II | 99 |
|  | c.5439G>C | p.E1813D | tumor-specific | PPB type II | 89 |
|  | c.5439G>C | p.E1813D | tumor-specific | PPB type II | 107 |

Abbreviations: CN cystic nephroma; PPB pleuropulmonary blastoma; SIP small intestine polyp; SLCT Sertoli-Leydig cell tumor; TCa thyroid carcinoma

**Table S8. Sequencing results from children with mosaic *DICER1* loss-of-function mutations**

| Study  ID | Tissue  Source | Tumor puritya | *DICER1* LOF mutation  (NM_177438.2) | LOF allele frequency  (variant / total reads)b | RNase IIIb domain  Hotspot mutation | Hotspot allele frequency  (variant / total reads)b |
| --- | --- | --- | --- | --- | --- | --- |
| 89 | Saliva | – | c.1165_1174dupCCATATGAGC;  p.R392Pfs3* | 1.1% (26/2273) | ND | ND |
|  | Fibroblasts | – | c.1165_1174dupCCATATGAGC;  p.R392Pfs3* | 8.10% (538/6646) | ND | ND |
|  | PPB Type II | 90% | c.1165_1174dupCCATATGAGC;  p.R392Pfs3* | 21% (20/91) | c.5439G>C; p.E1813D | 49% (46/91) |
| 90 | Blood | – | c.4426_4427insT; p.D1476Vfs5* | 4.7% (91/1949) | ND | ND |
|  | PPB Type III | 80% | c.4426_4427insT; p.D1476Vfs5* | 21.5% (200/929) | c.5424G>A; p.G1809R | 48% (476/993) |
| 5 | Blood | – | c.745C>T; p.Gln249* | 1.79% (177/9911) | ND | ND |
|  | PPB Type I | 10% | c.745C>T; p.Gln249* | 14.5% (290/1994) | c.5125C>T; p.D1709N | 9.8% (40/408) |
| 92 | Blood | – | c.2236A>G;p.R746G | 3.5% (70/1997) | ND | ND |
|  | PPB Type III | NA | NA | NA | NA | NA |
| 93 | Blood | – | c.1716delT; p.F572Lfs*15 | 17.2% (341/1977) | ND | ND |
|  | PPB Type I | NA | NA | NA | NA | NA |

Abbreviations: LOF loss-of-function; ND none detected; NA not available; PPB pleuropulmonary blastoma.

a. Approximate percent tumor cells in specimen, estimated visually by microscopy in tumor sections.

b. Allele frequency estimates derived from NGS read counts.

**Table S9. Sequence results from children with tumor-specific, biallelic *DICER1* mutations**

| Study  ID | Tissue  source | Tumor puritya | *DICER1* LOF mutation  (NM_177438.2) | LOF allele frequency  b  (variant / total reads)  {informative SNP} | RNase IIIb domain  Hotspot mutation | Hotspot allele frequency  (variant / total reads)b |
| --- | --- | --- | --- | --- | --- | --- |
| 91 | Blood | – | ND | {rs11624081; 49.8%} | ND | – |
|  | PPB Type II | 99% | ND; allele loss | {rs11624081; 0.0%} | c.5125C>T; p.D1709N | ~ 90% (37/41) |
| 94 | Blood | – | ND | – | ND | – |
|  | PPB Type III | 99% | c.1525C>T; p.R509* | 45.3% (904/1994) | c.5424G>A; p.G1809R | 49% (967/1999) |
| 95 | Blood | – | ND | – | ND | – |
|  | PPB Type I | 40% | c.996C>A; p.Y332* | 40.9% (606/1479) | c.5428G>T; p.D1810Y | 34.9% (389/1115) |
| 96 | Blood | – | ND | – | ND | – |
|  | PPB, Type III | 99% | ND; allele loss | – | c.5438A>G; p.E1813G | 96% (1577/1640) |
| 97 | Blood | – | ND | – | ND | – |
|  | PPB Type III | 15% | c.3540C>A; p.Y1180* | 58.8% (4212/7164) | c.5113G>A; p.E1705K | 25.3% (173/683) |
| 98 | Blood | – | ND | – | ND | – |
|  | PPB Type III | 99% | ND; allele loss | – | c.5425G>A; p.G1809R | 99% (1286/1303) |
| 99 | Blood | – | ND | – | ND | – |
|  | PPB Type II | 99% | ND; allele loss | – | c.5438A>G; p.E1813G | 99% (1923/1998) |
| 100 | Blood | – | ND | – | ND | – |
|  | PPB Type III | 99% | ND; allele loss | – | c.5428G>A; p.D1810Y | 93% (1852/1992) |
| 106 | Blood | – | ND | – | ND | – |
|  | PPB Type II | 40% | c.2026C>T; p.R676* | 36.7% (731/1993) | c.5425G>A; p.G1809R | 24.8% (496/2000) |
| 107 | Blood | – | c.832C>T; p.L278F | 0.17% (14/8001) | ND | – |
|  | PPB Type II | 20% | c.832C>T; p.L278F | 30.6% (611/1998) | c.5439G>C; p.E1813D | 26.9% (533/1984) |
| 111 | Blood | – | ND | {rs66997818; 50%} | ND | – |
|  | PPB Type II | 50% | c.5104C>T; p.Q1702*  accompanied by allele loss | ~ 6% (4/72)  {rs66997818; 97.8%} | c.5125C>T; p.D1709N | ~ 47% (34/73) |
| 112 | Blood | – | ND | {rs61751177; 46.8%} | ND | – |
|  | PPB Type II | 60% | ND; allele loss | {rs61751177; 79.8%} | c.5125G>A; p.D1709N | 62.5% (208/333) |

Abbreviations: LOF loss-of-function; ND none detected; PPB pleuropulmonary blastoma.

a. Approximate percent tumor cells in specimen, estimated visually by microscopy in tumor sections.

b. Allele frequency estimates derived from NGS read counts.

**Table S10. Unresolved cases: PPB children with no predisposing *DICER1* mutation identified**

| Study ID | PPB type | Other syndromic disease | Total # of disease foci | Affected family members | Survival status alive=1 dec’d=0 | Germline mutation testing | | | | Tumor testing |
| --- | --- | --- | --- | --- | --- | --- | --- | --- | --- | --- |
|  |  |  |  |  |  | *DICER1* amplicon NGS | NanoString copy number | Exome seq.a | Additional miRNA pathway genesb | *DICER1* amplicon NGS: mutation;  allele frequencyc  (read counts) |
| 113 | III | unknown | 1 | no | 1 | ND | normal | ND | ND | NA |
| 114 | II | none | 1 | no | 1 | ND | NT | ND | ND | NA |
| 115 | II | none | 1 | no | 1 | ND | PQ | NT | ND | NA |
| 116 | I | none | 1 | no | 1 | ND | normal | NT | ND | NA |
| 117 | III | unknown | 1 | no | NA | ND | PQ | NT | ND | NA |
| 118 | I | none | 1 | no | 1 | ND | normal | NT | ND | NA |
| 119 | II | none | 1 | no | 0 | ND | normal | ND | ND | NA |
| 121 | I | none | 1 | no | 1 | ND | normal | ND | ND | c.5125G>A; p.D1709N;  PPB: ~13% (10/74) |
| 122 | Ir | none | 1 | no | 1 | ND | PQ | ND | ND | PQ |
| 124 | II | none | 1 | no | 1 | ND | NT | ND | ND | c.5113G>A; p.E1705K  PPB: ~40% (3/7) |

Abbreviations: CN cystic nephroma; NA not available; ND none detected; NT not tested; PPB pleuropulmonary blastoma; PQ poor quality (not interpretable); SNP single nucleotide polymorphism.

a. Exome sequencing (Illumina) from blood DNA.

b. Sanger sequencing in blood DNA for *DROSHA*, *XPO5*, and the *DICER1* promoter region.

c. Allele frequency estimates derived from NGS read counts.

Note: This data set is limited, pursuant to concerns for potential identification of study participants based on particular combinations of clinical and pathologic features. Qualified investigators with specific questions about the study not answered by the data in these tables are invited to contact the International PPB Registry. The Registry will try to accommodate requests for additional data while preserving protected health information.
