# Supplementary material for: Temporal order of RNase IIIb and loss-of-function mutations during development determines phenotype in pleuropulmonary blastoma / DICER1 syndrome: a unique variant of the two-hit tumor suppression model
Source: F1000Res. 2018 Jan 12;4:214. Originally published 2015 Jul 10. [Version 2] doi: 10.12688/f1000research.6746.2 (PMC4712775; doi:10.12688/f1000research.6746.2)
Supplement: Peer review history

# Peer review: Referee response for version 2

**DOI:** 10.5256/f1000research.14447.r29770

**Published:** 2018-04-27

**Authors:** James F. Amatruda, Kenneth S. Chen

**Affiliations:** 1 Departments of Pediatrics, Molecular Biology and Internal Medicine, University of Texas Southwestern Medical Center, Dallas, TX, USA; 2 Department of Pediatrics, University of Texas Southwestern Medical Center, Dallas, TX, USA

The revised manuscript published 12 Jan 2018, entitled “Temporal order of RNase IIIb and loss-of-function mutations during development determines phenotype in pleuropulmonary blastoma/ *DICER1* syndrome: a unique variant of the two-hit tumor suppression model”, provides a significant insight into the correlation between genotype and phenotype in DICER1 syndrome patients. This work will contribute to the growing base of literature to inform the decisions being made as guidelines are being written regarding testing and tumor surveillance for these patients. 

 Recent work from this group and others has demonstrated the occurrence of two distinct types of *DICER1*mutations in human tumors: 1) nonsense or frameshift mutations predicted to create a null allele; and 2) “hotspot” mutations occurring on or adjacent to acidic metal-binding residues in the Ribonuclease IIIB domain of DICER1. Here, Brenneman et al. use the large sample size of the International PPB Registry to show that there are in fact three ways to develop a compound heterozygous (null plus hotspot) *DICER1*tumor, in decreasing order of frequency: a germline null with a somatic hotspot; a germline mosaic hotspot with a somatic null; or two somatic mutations. However, they provide the significant insight that each of these categories corresponds to a unique clinical severity. In particular, patients with a germline mosaic hotspot appear to develop the most severe disease, presumably because inactivation of the wildtype allele via nonsense or frameshift is a more frequent event than a hotspot mutation. 

                                                                                                                                         

We have read this submission. We believe that we have an appropriate level of expertise to confirm that it is of an acceptable scientific standard.

# Peer review: Referee response for version 2

**DOI:** 10.5256/f1000research.14447.r29771

**Published:** 2018-02-02

**Authors:** Julian A. Martinez-Agosto, Steven Klein

**Affiliations:** 1 Departments of Pediatrics and Human Genetics, David Geffen School of Medicine, University of California, Los Angeles, Los Angeles, CA, USA

We are delighted that the authors pursued additional genetic characterization of unsolved cases and confirmed the presence of mosaic RNASEIIIb domain mutations hotspot mutations, further demonstrating the somatic distribution of these variants and their distinct syndromic presentation. Furthermore, the revised discussion of alternate models of tumorigenesis is representative of the unique tumor suppresor and oncogenic roles of *DICER1 *mutations. This study is an invaluable resource that establishes the genotype-phneotype spectrum for *DICER1*-related disorders and serves as the basis for additional studies into the mechanism of action of pathogenic mutations.

We have read this submission. We believe that we have an appropriate level of expertise to confirm that it is of an acceptable scientific standard.

# Peer review: Referee response for version 1

**DOI:** 10.5256/f1000research.7245.r10861

**Published:** 2015-10-19

**Authors:** James F. Amatruda, Kenneth S. Chen

**Affiliations:** 1 Departments of Pediatrics, Molecular Biology and Internal Medicine, University of Texas Southwestern Medical Center, Dallas, TX, USA; 2 Department of Pediatrics, University of Texas Southwestern Medical Center, Dallas, TX, USA

In this manuscript, entitled “Temporal order of RNase IIIb and loss-of-function mutations during development determines phenotype in *DICER1* syndrome: a unique variant of the two-hit tumor suppression model”, Brenneman *et al*. present a comprehensive, state-of-the-art analysis of the various types of *DICER1* mutations seen in patients with pleuropulmonary blastoma and other *DICER1*-related tumors. In their tumors, these patients often display a combination of a loss-of-function mutation on one allele of *DICER1 *and a hotspot RNase IIIb missense mutation on the opposite allele. As the authors show, tumors may acquire *DICER1* mutations via germline inheritance, post-zygotic mosaicism, or somatic mutation at the time of tumor formation, and the mutations can be acquired in either order. Importantly, patients can be categorized based on the status of their inherited *DICER1* alleles (wild-type, loss-of-function or hotspot missense; and germline or mosaic), and this categorization determines patient phenotype.

                                             

This is a timely report, given the recent surge of reports implicating *DICER1* mutations in human cancers. The authors present a robust clinical and molecular characterization of a large cohort of PPB patients. The work is of high quality and the report is clearly written. The conclusions are largely supported by the data, with some possible exceptions outlined below. The large amount of supplementary data is a particularly valuable resource and serves as a model for studies of this kind. Overall this is a very valuable contribution to the literature on *DICER1*-related cancers. The reservations noted center a few remaining areas of ambiguity concerning the molecular model presented by the authors.

**Title**

1. *“Temporal order of RNase IIIb and loss-of-function mutations during development determines phenotype in DICER1 syndrome: a unique variant of the two-hit tumor suppression model”*: This study does not appear to investigate *DICER1* syndrome *per se*, but rather PPB. Inclusion criteria was “PPB patients (n=124)”, and this includes 12 patients who were found to have no germline or mosaic *DICER1 *mutation (Table S8) and another 12 patients who had no detectable *DICER1* mutations (Table S9).

**Introduction**

1. *PPB is pathognomonic for a childhood cancer syndrome that features a range of other benign and malignant neoplasms including ovarian Sertoli-Leydig cell tumor (SLCT), …” * As many of the *DICER1*-related cases of SLCT that have been reported occur in patients in their 30s and 40s (See for example Rio Frio *et al.,* 2011), it is probable that *DICER1* syndrome is not simply a “childhood” cancer syndrome.
2. *“Understanding how the interplay of RNase IIIb missense and LOF mutations influences the expression of syndromic neoplasias can aid diagnosis at early stages, when they are most curable.”*  As far as we know there are no data that early diagnosis of *DICER1*-syndrome neoplasias (other than, potentially, PPB) is beneficial.  In fact, in this manuscript, the patients with mosaic hotspot mutations presented with lower-type PPB (non-sarcomatous) but underwent much more “complicated and arduous” clinical courses.
3. *“We propose that the extreme phenotypes of this patient group are attributable to the order in which allelic DICER1 mutations were acquired during development, i.e., an RNase IIIb hotspot missense mutation acquired early in embryogenesis and subsequently unmasked by LOF mutations or loss of the second allele.” *The authors’ model (biallelic mutations are fundamental to the development of *DICER1*-related tumors, hence the need for a loss/LOF mutation in trans to a hotspot missense mutation) may not be universally true, or may be true for PPB but not other *DICER1*-realted tumors. While the frequency of biallelic *DICER1* mutations appears to be high in PPB (this study; Pugh *et al *[Ref.6]; Seki *et al *[Ref. 35]), this does not appear to be the case in all *DICER1*-realted tumors.  We previously reported a Wilms tumor patient with a DICER1 hotspot missense mutation and no detected germline mutation, though we did not rule out copy-number loss of the wildype allele ( Rakheja *et al., *2014). And the TARGET sequencing project reported *DICER1* variants in Wilms tumor patients, including two patients who demonstrated  non-hotspot germline missense mutations but no mutations on the other allele in their tumors ( Walz *et al.*, 2015).

**Methods**

1. *“Definition of ‘disease foci’”*: Several of the “disease foci” in Table 1 are left off this list (e.g., Wilms tumor, juvenile polyps of small intestine, ciliary body medullopeithelioma).  Was this list dynamically expanded during the course of the study?

**Results**

1. What is the SIFT score for I582T?
2. *“no conclusive evidence of the variants in parental blood”*: What was considered “conclusive evidence”? What read depth was obtained in these parents?
3. *“Preliminary data from an ongoing NCI-sponsored DICER1 family history study”*: Understanding that these are unpublished data, it would be helpful if the authors could state approximately how large is the sample size. Do all asymptomatic patients in this study undergo thyroid ultrasound and lung CT?
4. *“We categorized a DICER1 mutation detected by NGS as mosaic when the following criteria were met: i. The mutation was evidently not a constitutional, germline allele because it was present at sub-heterozygous frequency (arbitrarily taken as below 35% of reads)*”: According to Table S7, even in the two cases where tumor purity was 80-90%, % of reads supporting the loss-of-function allele was only 21%.  This argues that either 35% is too high of a cutoff for determining subtotal mosaicism, or the tumors do not possess this mutation in every cell.  Could the authors speculate on which is more likely?
5. Table 2: The cystic nephroma in patient 103 and the Wilms tumor in Klein Case 1 both feature missense mutations in the non-hotspot allele (p.V377I and p.P453L, respectively, and the effect if any of these mutations on DICER1 activity is not known. Thus it may be premature to label these cases truly two-hit in nature.
6. The fact that some tumors in children with mosaic hotspot mutations acquire several different LOF mutations (such as Study ID 103) agrees with the model. However the allele frequencies are overall low (3-4% in this tumor) making it unclear how significant the LOF event actually was.
7. The authors  may also want to consider the possibility that, in cells with a hotspot mutation and an intact WT DICER1 allele, 5p/3p miRNA skewing leads to defects in DNA replication or repair, predisposing to a second hit. Such a mechanism could in theory help explain the higher incidence of tumors in this group of patients, along with the fact that many more codons of *DICER1* may be mutated to casue a LOF allele, compared with the hotspot missense mutations.
8. Table 2: how was “allele loss” determined?
9. Figure 3 is confusing, as pt 105 appears to be on a different x-axis than the other four patients

**Discussion**

1. *“Additionally, we hypothesize that in mosaic hotspot children, wider tissue/organ distribution of aberrant miRNA processing during development may produce syndromic features not seen in children with predisposing LOF mutations, such as juvenile-type small intestinal polyps”*: Could the authors speculate on why hotspot mutations would cause small intestinal polyps but LOF mutations would not? In the one case (#102, table 2) in which a polyp was sequenced, it was found to harbor both a LOF and hotspot mutation. As the hotspot mutation is the rate-limiting step, it seems more likely that LOF children also develop polyps, but at a lower frequency, and that their small intestines have not been thoroughly examined for the presence of polyps.
2. *“It may be impossible to unequivocally rule out mosaicism, but techniques such as targeted resequencing by high depth NGS in multiple tissues can greatly improve diagnostic confidence”*: Usually, it is clinically unfeasible to perform high-depth NGS in multiple tissues from a patient. In patients whose germline *DICER1 *sequencing is negative, it may be more helpful to use clinical proxies to identify patients at high risk for mosaicism, such as young age and multifocal disease.

**Other**

1. Throughout the manuscript, there are a few instances where “RNAse IIIb” is used instead of “RNase IIIb”. The latter is more standard nomenclature.

We have read this submission. We believe that we have an appropriate level of expertise to confirm that it is of an acceptable scientific standard, however we have significant reservations, as outlined above.

# Response

**Published:** 2018-01-11

**Authors:** D. Ashley Hill

**Affiliations:** Children's National Medical Center, USA

**Author Responses in Bold to Referee Repor**t

 James F. Amatruda, Departments of Pediatrics, Molecular Biology and Internal Medicine, University of Texas Southwestern Medical Center, TX, USA 

 Kenneth S. Chen, Department of Pediatrics, University of Texas Southwestern Medical Center, USA 

**Approved with Reservations**

 In this manuscript, entitled “Temporal order of RNase IIIb and loss-of-function mutations during development determines phenotype in *DICER1* syndrome: a unique variant of the two-hit tumor suppression model”, Brenneman *et al*. present a comprehensive, state-of-the-art analysis of the various types of *DICER1* mutations seen in patients with pleuropulmonary blastoma and other *DICER1*-related tumors. In their tumors, these patients often display a combination of a loss-of-function mutation on one allele of *DICER1 *and a hotspot RNase IIIb missense mutation on the opposite allele. As the authors show, tumors may acquire *DICER1* mutations via germline inheritance, post-zygotic mosaicism, or somatic mutation at the time of tumor formation, and the mutations can be acquired in either order. Importantly, patients can be categorized based on the status of their inherited *DICER1* alleles (wild-type, loss-of-function or hotspot missense; and germline or mosaic), and this categorization determines patient phenotype.

                                              

 This is a timely report, given the recent surge of reports implicating *DICER1* mutations in human cancers. The authors present a robust clinical and molecular characterization of a large cohort of PPB patients. The work is of high quality and the report is clearly written. The conclusions are largely supported by the data, with some possible exceptions outlined below. The large amount of supplementary data is a particularly valuable resource and serves as a model for studies of this kind. Overall this is a very valuable contribution to the literature on *DICER1*-related cancers. The reservations noted center on a few remaining areas of ambiguity concerning the molecular model presented by the authors.

**Title**

*“Temporal order of RNase IIIb and loss-of-function mutations during development determines phenotype in DICER1 syndrome: a unique variant of the two-hit tumor suppression model”*: This study does not appear to investigate *DICER1* syndrome *per se*, but rather PPB. Inclusion criteria was “PPB patients (n=124)”, and this includes 12 patients who were found to have no germline or mosaic *DICER1 *mutation (Table S8) and another 12 patients who had no detectable *DICER1* mutations (Table S9).

      

**The title has been revised to reflect our study’s emphasis on PPB:**

****

***Temporal order of RNase IIIb and loss-of-function mutations during development determines phenotype in pleuropulmonary blastoma/ DICER1 syndrome: a unique variant of the two-hit tumor suppression model***

****

**We strove to survey the spectrum of pediatric neoplasias now associated with *DICER1* mutations, as reflected by the summary of diverse organ sites involved in Table 1. As you surmised, this project began as a prospective study of predisposing *DICER1* mutations in PPB. Associations of other tumor types with *DICER1* mutation and the concept of an overarching *DICER1* syndrome emerged over the study’s course. In practice, PPB remained a good inclusion criterion for a study of *DICER1* syndrome because nearly all PPB is associated with *DICER1* mutation, though as it turns out, not always germline mutation. For other tumors in the *DICER1* spectrum, such as SLCT, the association is less constant and for some, *e.g.*, Wilms tumor, it is rare. Moreover, it appears that most pediatric patients who present with *DICER1* spectrum neoplasias of any kind, and are then confirmed to carry a germline or mosaic *DICER1* mutation, have some degree of lung involvement even where PPB or lung cysts were not the first finding. If starting the study today, we would include patients who present with other syndromic neoplasias and no lung involvement. We have encountered some. Unfortunately, cases of that kind were not accrued prospectively or with as much supporting data.**

**The 12 PPB children previously listed in Table S8 (now Table S9 in the revised supplemental tables) were categorized as having biallellic *DICER1* mutations specific to the tumor, but this is a laboratory distinction – a reflection of what we could detect, not necessarily the underlying biology. They may represent a more limited form of mosaicism, *i.e.*, mutations that occurred late in prenatal development and have a correspondingly restricted tissue distribution – perhaps so restricted that it gave rise to only a single focus of PPB in one lung.**

**The 12 cases previously listed in Table S9 (now Table 10) should not be regarded as cases of PPB *with no detectable DICER1 mutations*. They were unresolved cases – ones we could not catagorize at that time. We have since confirmed two cases, study ID# 123, as an RNase IIIb hotspot mosaic (please see Table 2 in the revised manuscript). The Foulkes lab has since published results for study ID#120, indicating this patient is also a RNase IIIb hotspot mosaic, and we have some data supporting this. In nine of the ten remaining cases, tumor specimens are unavailable or too poorly preserved to sequence. *DICER1* mutations have not been excluded in the tumors of those nine patients. The presence of cryptic mosaic or germline *DICER1* mutations has been not been excluded for any of the ten patients. As this was a prospective study, we think it best to account for all the accrued PPB patients in this report, rather than omit some because of unresolved *DICER1* mutation status.**

**Introduction**

*1. PPB is pathognomonic for a childhood cancer syndrome that features a range of other benign and malignant neoplasms including ovarian Sertoli-Leydig cell tumor (SLCT), …” * As many of the *DICER1*-related cases of SLCT that have been reported occur in patients in their 30s and 40s (See for example *Rio Frio et al., 2011*), it is probable that *DICER1* syndrome is not simply a “childhood” cancer syndrome.

**The first paragraph of the Introduction has been revised to mention occasional diagnoses in adults. With regard to *Rio Frio 2011*, “ *many*” may be a little too strong. One of five SLCTs in *DICER1* mutation carriers described in that report was diagnosed at 32 years. The other four were diagnosed at ages in the teens or not specified. Table 2 of *Rio Frio 2011* summarizes previously published descriptions of familial SLCT patients including several older adults, but those are much earlier papers and *DICER1* mutation wouldn’t have been looked for. Similarly, *Shultz et al 2012* identified 8 patients with germline *DICER1* mutations and SLCT or other ovarian sex cord stromal tumors. One patient was diagnosed at 32 years; the others at ages ranging from 2 to 18 years. The larger question of whether and to what extent germline *DICER1* mutations might figure in new onsets of neoplastic disease in adulthood is of great interest, but answers may have to await longer follow-up in identified families.**

 2. *“Understanding how the interplay of RNase IIIb missense and LOF mutations influences the expression of syndromic neoplasias can aid diagnosis at early stages, when they are most curable.”* As far as we know there are no data that early diagnosis of *DICER1*-syndrome neoplasias (other than, potentially, PPB) is beneficial. In fact, in this manuscript, the patients with mosaic hotspot mutations presented with lower-type PPB (non-sarcomatous) but underwent much more “complicated and arduous” clinical courses.

      

**Certainly the degree of benefit from earlier diagnosis will vary among *DICER1* syndromic tumor types, as some have greater potential for malignant progression than others. For PPB, the benefits of early diagnosis are unequivocal. But rather than embark on a detailed breakdown in the Introduction, we have removed the phrase “ *when they are most curable*”.**

**With regard to the mosaic hotspot patients in this cohort, two had type II PPB and one has since died of metastatic disease. It’s important to remember the hotspot mosaic patients were recognized as such only in retrospect, after the analysis described in this report. Their clinical course was difficult, despite earlier diagnosis with (mostly) lower-type PPB, because of additional tumors in multiple organ sites. We still have no way to prevent that. But going forward, it will at least be possible to recognize hotspot mosaicism when present in children with PPB and/or other *DICER1* syndromic tumors, and to anticipate exceptionally high risk for additional neoplastic disease. This improves our ability to educate caregivers and primary-care physicians about what to expect and watch for.**

*3. “We propose that the extreme phenotypes of this patient group are attributable to the order in which allelic DICER1 mutations were acquired during development, i.e., an RNase IIIb hotspot missense mutation acquired early in embryogenesis and subsequently unmasked by LOF mutations or loss of the second allele.” *The authors’ model (biallelic mutations are fundamental to the development of *DICER1*-related tumors, hence the need for a loss/LOF mutation in trans to a hotspot missense mutation) may not be universally true, or may be true for PPB but not other *DICER1*-related tumors. While the frequency of biallelic *DICER1* mutations appears to be high in PPB (this study; Pugh *et al *[Ref.6]; Seki *et al *[Ref. 35]), this does not appear to be the case in all *DICER1*-realted tumors.  We previously reported a Wilms tumor patient with a *DICER1* hotspot missense mutation and no detected germline mutation, though we did not rule out copy-number loss of the wild type allele ( *Rakheja et al., 2014*). The TARGET sequencing project also reported *DICER1* variants in Wilms tumor patients, including two patients who demonstrated  non-hotspot germline missense mutations but no mutations on the other allele in their tumors ( *Walz et al., 2015*).

      

**The model we propose is an attempt to explain (in part) why children with *DICER1* mutations differ so dramatically with respect to numbers of disease foci and organ sites involved, and particularly the role of mosaicism. We share your view that a hotspot-plus-LOF model may not apply to all *DICER1*-associated tumors in all organ sites, especially with regard to some of the rarer manifestations of *DICER1* syndrome – Wilms tumor, non-epithelial ovarian tumors and pineoblastoma particularly. We have extended the Discussion to address this.**

**Methods**

 1. *“Definition of ‘disease foci’”*: Several of the “disease foci” in Table 1 are left off this list (e.g., Wilms tumor, juvenile polyps of small intestine, ciliary body medullopeithelioma). Was this list dynamically expanded during the course of the study? 

      

**An oversight – thank you. The description in Methods now includes all neoplasias shown in Table 1.**

**Results**

 1. What is the SIFT score for I582T? 

      

**The SIFT score for I582T was 0.0, compared to a median of 3.55 for other substitutions at the analogous position in related genes of 11 species. It was classified as deleterious but of unknown pathogenicity. We have expanded Table S4 to show SIFT and PROVEAN results for all of the five germline (non-hotspot) missense mutations we found.**

 2. *“no conclusive evidence of the variants in parental blood”* What was considered conclusive evidence? What read depth was obtained in these parents?

      

**The putative *de novo* mutations in question were all single nucleotide variants, which can also occur as sequencing errors. In sequencing data from parental blood, “mutant” reads occuring at frequencies close to the predicted error frequency of the Ion Torrent platform were not taken as conclusive evidence of parental mosaicism. A new supplemental table, Table S5, summarizes the parental sequencing results, including read depths.**

 3. *“Preliminary data from an ongoing NCI-sponsored DICER1 family history study”*: Understanding that these are unpublished data, it would be helpful if the authors could state approximately how large is the sample size. Do all asymptomatic patients in this study undergo thyroid ultrasound and lung CT?

**Thyroid ultrasound and lung CT are offered to all identified *DICER1* mutation carriers, but perhaps not all accept. Of *48* asymptomatic carriers so far screened by CT, *12* have lung cysts. The part of the study concerned with thyroid disease would be harder to summarize here, but has now been published: Khan et al, J. Clin. Endocrinol. Metab. 2017 May 1;102(5):1614-1622. doi: 10.1210/jc.2016-2954. PMID: 28323992**

 4. *“We categorized a DICER1 mutation detected by NGS as mosaic when the following criteria were met: i. The mutation was evidently not a constitutional, germline allele because it was present at sub-heterozygous frequency (arbitrarily taken as below 35% of reads)*”: According to Table S7, even in the two cases where tumor purity was 80-90%, the percentage of reads supporting the loss-of-function allele was only 21%.  This argues that either 35% is too high of a cutoff for determining subtotal mosaicism, or the tumors do not possess this mutation in every cell. Could the authors speculate on which is more likely?

      

**That part of the sentence has been corrected to read “... *because it was present at sub-heterozygous frequency in blood or normal tissue specimens (arbitrarily taken as below 35% of reads)*”.  No cutoff was applied for tumor specimens. But your point about allele frequencies in tumors of LOF mosaic patients is still valid. In the PPB tumor specimens of patients 89 and 90, allele frequencies of the detected LOF mutations are only about half of that expected for specimens having 80-90% tumor purity (revised supplemental Table S8). Even with allowance for inexact visual estimates of tumor purity, the conclusion seems inescapable: the detected LOF mutation is not present in every tumor cell. There is a similar instance in hotspot mosaic patient 111 (please see revised Table S9). Our working hypothesis is that the “missing” LOF alleles in these tumors are exactly that – *deleted*. For patient 111, an informative SNP marker linked to the *DICER1* locus was also genotyped and the results support allele loss in the tumor. Moreover, there is evidence of allele loss in seven of the twelve sporadic PPB specimens in revised Table S9, which suggests some level of genome instability prevails in these tumors.**

 5. Table 2: The cystic nephroma in patient 103 and the Wilms tumor in Klein Case 1 both feature missense mutations in the non-hotspot allele (p.V377I and p.P453L, respectively) and the effect if any of these mutations on DICER1 activity is not known. Thus it may be premature to label these cases truly two-hit in nature.

      

**Yes, it may be. The only other mutations in our data set resembling these two are the I582T germline mutation in patient 36 (noted in item 1, above) and the L278F mutation in patient 107 (detected in a PPB tumor and at very low level in blood). Like V377I and P453L, these missense mutations are in or near the N-terminal helicase domain of DICER1, the functional importance of which is uncertain. An additional missense mutation, R746G, lying between the helicase and PAZ domains, was found at low allele frequency in the blood of patient 92 (revised Table S8). This was classified as a mosaic LOF allele solely on the basis of its presence in a child with PPB (type III; unfortunately not available for sequencing). To assist readers in coming to their own conclusions, additional annotation is provided for each of the missense mutations in revised Table S4.**

 6. The fact that some tumors in children with mosaic hotspot mutations acquire several different LOF mutations (such as Study ID 103) agrees with the model. However the allele frequencies are overall low (3-4% in this case), making it unclear how significant the LOF event actually was.

****

**Yes, if we assume there was only the detected LOF event and no other. The confounding problem is that we have not ruled out cryptic LOF mutations or allele losses not readily detected by the methods we used. It’s fairly straightforward to show that a nonsense or frameshift mutation is present or not, as they must occur in coding sequence. But cryptic LOF mutations could be any kind of event from single nucleotide changes in non-coding regulatory sequences to chromosomal rearrangements that preserve all wild type coding sequence but de-link the exons. Allele loss can be difficult to establish on the basis of read counts in specimens of low tumor purity.**

 7. The authors may also want to consider the possibility that, in cells with a hotspot mutation and an intact WT *DICER1* allele, 5p/3p miRNA skewing leads to defects in DNA replication or repair, predisposing to a second hit. Such a mechanism could in theory help explain the higher incidence of tumors in this group of patients, along with the fact that many more codons of *DICER1* may be mutated to cause an LOF allele, compared with the hotspot missense mutations.

      

**We’re very interested in that possibility too, but chose not to speculate, because we had no data showing 5p/3p miRNA skewing in tumors or tissues with one hotspot allele and one wild type. The best and only data of that kind we know about is your own. In Rakheja *et al* 2014, Figure 3d shows clear skewing of 5p/3p miRNA output from Wilms tumor specimen CMCW59, in which only a G1809V hotspot mutation was detected. Although skewing is not so extreme as in CMCW11 (G1809R + frameshift LOF), it still amounts to inversion of the characteristic 5p/3p ratio seen in wild type specimens, and it’s hard to imagine this has no consequence for gene regulation. A related possibility is that *DICER1* RNase IIIb hotspot mutations exert a dominant negative effect over wild type *DICER1*, as you demonstrated for RNase IIIb mutation in *DROSHA*. What the mechanism of a dominant negative effect on miRNA processing by mutant DICER1 might be is unclear.**

**A difficulty of interpretation for tumors like CMCW59 or tumors of patients 120, 123 and 124 in this study, in which only a hotspot muatation has been detected, is to rule out cryptic LOF mutation. It’s hard to be certain whether a tumor’s genotype is truly *DICER1*^hotspot/wildtype^ or actually *DICER1*^hotspot/LOF^. Perhaps the best way to model effects of a *DICER1*^hotspot/wildtype^ genotype on miRNA processing and gene expression would be a genome editing approach like that used in Rakheja *et al* 2014 for *DROSHA.***

**Effects of *DICER1* hotspot mutations on genome stability might well arise through 5p/3p miRNA skewing. Over thirty genes related to DNA repair or replication have shown altered expression levels in PPB tumors (Hill *et al*, unpublished). But in thinking about genome stability and mutagenesis, we also wonder whether catalytic activities of DICER1 (or DROSHA) might figure in other nucleic acid transactions besides miRNA processing. Could they have more direct roles in replication or repair?**

 8. Table 2: how was “allele loss” determined? 

      

**Allele loss was inferred from allele frequency disparities. In eight separate tumors arising in four hotspot mosaic patients (102, 104, 105 and 123), no specific LOF coding sequence change was detected and relative frequency of the hotspot missense allele is equal to or greater than the estimated tumor purity of the specimen. This implies that all or nearly all copies of *DICER1* present in tumor cells are the hotspot missense allele, *i.e.*, the second allele has been lost in most tumor cells. In the example calculations that follow, “wild type” means only that no mutation was present in amplicons representing exons 24 or 25, where the hotspot codons are located; an LOF mutation could be present elsewhere in the gene.**

**Example 1. In the left ovarian SLCT of patient 104, an estimated 95% of cells in the specimen are tumor cells and we assume they all carry the hotspot missense allele. Let 2 *n* be the total number of *DICER1* alleles present in a sample of tissue or tumor comprising n diploid cells. Consider three hypothetical allele distributions:**

****

**A. If both a hotspot missense mutant allele and a wild type allele are present in all tumor cells, and two wild type alleles are present in non-tumor cells making up the remaining 5% of the specimen, then:**

- **Hotspot missense allele frequency would be: (.95 x 1n) / 2n = 47.5%**
- **Wild type allele frequency would be: (.95 x 1n + .05 x 2n) / 2n = 1.05n / 2n = 52.5%**

**B. However, if all tumor cells are hemizygous at *DICER1*, retaining only the hotspot missense allele, and all non-tumor cells are homozygous wild type, then the total number of *DICER1* alleles present in the specimen would be reduced from 2n to only 1.05n, *i.e.,* 0.95 x 1n + 0.05 x 2n.**

- **Hotspot missense allele frequency would then be (.95 x 1n) / 1.05n = 90.5%**
- **Wild type allele frequency would be (.05 x 2n) / 1.05n = 9.5%**

**C. Alternately, if all tumor cells are hemizygous but the 5% non-tumor cells are all heterozygous (one missense allele and one wild type), then:**

- **Hotspot missense allele frequency would be (.95 x 1n + .05 x 1n) / 1.05n = 95.2%**
- **Wild type allele frequency would be (.05 x 1n) / 1.05n = 4.8%.**

**      The actual allele frequencies measured in this specimen were 92.4% hotspot missense and 7.6% wild type. These frequencies are intermediate to those predicted in alternatives B and C above for tumor cell populations hemizygous at *DICER1* (having lost their wild-type allele). The ~ 5% non-tumor cells in this specimen might be accounted for as a mix of heterozygous (hotspot mutant /wild type) and homozygous wild type cells, consistent with somatic mosaicism.**

****

**Example 2. Patient 102, PPB brain metastasis: estimated tumor purity 30%**

**A. If both a hotspot missense mutant allele and a wild type allele is present in all tumor cells, and two wild type alleles are present in non-tumor cells making up the remaining 70% of the specimen, then:**

- **Hotspot missense allele frequency would be: (.30 x 1n) / 2n = .30n / 2n = 15%**
- **Wild type allele frequency would be: (.30 x 1n + .70 x 2n) / 2n = 1.7n / 2n = 85%**

**B. If all tumor cells are hemizygous at *DICER1* (having lost their wild-type allele) and all non-tumor cells are homozygous wild type, then the total number of *DICER1* alleles present in the specimen would be reduced from 2n to (0.30 x 1n) + (0.70 x 2n) = 1.70n, and:**

- **Hotspot missense allele frequency would then be (.30 x 1n) / 1.7n = 17.6%**
- **Wild type allele frequency would be (.70 x 2n) / 1.7n = 82.4%**

**C. Alternately, if all of the 30% tumor cells are hemizygous but the 70% non-tumor cells are all heterozygous (one missense allele and one wild type), then:**

- **Hotspot missense allele frequency would be (.30 x 1n + .70 x 1n) / 1.70n = 58.8%**
- **Wild type allele frequency would be (.70 x 1n) / 1.70n = 41.2%.**

**      The measured frequencies for this specimen were 51% hotspot mutation and 49% wild type. This appears most consistent with alternative C. Again, the non-tumor cells may be a mix of heterozygous and homozygous wild type cells, making the observed hotspot allele frequency somewhat lower than predicted and the wild type correspondingly higher.**

**Although other explanations for the allele frequencies seen in these tumors are certainly conceivable, allele loss appears the most straightforward.**

 9. Figure 3 is confusing, as patient 105 appears to be on a different x-axis than the other four patients. 

****

**In Figure 3, the clinical time line (x-axis) of patient 105 is interrupted at 7½ years (double slash symbol) and compressed to the right of that point so that events out to 17 years could be shown. This is a common graphical device, but admittedly less than ideal. We could alternatively show all patients on a longer, unbroken time scale, but that would require compressing the entire x-axis to fit the figure on a page. On balance, we thought it best to keep the interrupted timeline for patient 105 and preserve detail in the earlier years for all patients.**

**Discussion**

 1. *“Additionally, we hypothesize that in mosaic hotspot children, wider tissue/organ distribution of aberrant miRNA processing during development may produce syndromic features not seen in children with predisposing LOF mutations, such as juvenile-type small intestinal polyps”*: Could the authors speculate on why hotspot mutations would cause small intestinal polyps but LOF mutations would not? In the one case (#102, Table 2) in which a polyp was sequenced, it was found to harbor both a LOF and hotspot mutation. As the hotspot mutation is the rate-limiting step, it seems more likely that LOF children also develop polyps, but at a lower frequency, and that their small intestines have not been thoroughly examined for the presence of polyps.

      

**All the instances of juvenile intestinal polyps in mosaic hotspot children were discovered at surgery for intestinal intussusception. It may be that some children with germline or mosaic LOF mutations also developed juvenile intestinal polyps. If less numerous or smaller and not associated with intussusception, those might well escape notice, because examination of the small intestine for polyps has not been usual in diagnostic workup of children presenting with PPB or other *DICER1* syndromic tumors.**

**But we were alluding to the possibility you suggest in item 7 ( *Results*) above – that gene regulation is materially altered in cells bearing a mosaic hotspot mutation, *even while a wild type allele is retained*. In those embryonic tissues where a large fraction of stem or progenitor cells are *DICER1*^hotspot/wildtype^, some degree of 5p/3p skewing could conceivably have effects such as delayed cell lineage committment or incomplete differentiation, resulting in developmental disturbances, *e.g.*, exaggerated growth of localized tissue regions or entire organs.**

*2. “It may be impossible to unequivocally rule out mosaicism, but techniques such as targeted resequencing by high depth NGS in multiple tissues can greatly improve diagnostic confidence”*: Usually, it is clinically unfeasible to perform high-depth NGS in multiple tissues from a patient. In patients whose germline *DICER1 *sequencing is negative, it may be more helpful to use clinical proxies to identify patients at high risk for mosaicism, such as young age and multifocal disease.

      

**We were thinking of normal adjacent tissue recovered at tumor resection, together with sources that can be sampled without surgical biopsy, such as blood and buccal cells. In the future, development of digital droplet PCR assays for the limited spectrum of known RNase III hotspot mutations might eliminate any need for deep sequencing. But your point on clinical proxies is a good one and we have revised the paragraph. In a child who presents very early with multifocal disease, hotspot mosaicism should be suspected even if specimens for high-depth NGS are not available. More problematic is the child who presents early with a single, *DICER1*-mutant tumor and tests negative for germline mutation. Is this child at risk for additional tumors or not?**

**Other**

 1. Throughout the manuscript, there are a few instances where “RNAse IIIb” is used instead of “RNase IIIb”. The latter is more standard nomenclature.

**Corrected – thank you.**

# Peer review: Referee response for version 1

**DOI:** 10.5256/f1000research.7245.r9442

**Published:** 2015-07-24

**Authors:** Julian A. Martinez-Agosto, Steven Klein

**Affiliations:** 1 Departments of Pediatrics and Human Genetics, David Geffen School of Medicine, University of California, Los Angeles, Los Angeles, CA, USA

Brenneman *et al. *present an observational study on a cohort of patients with DICER1 Syndrome. The analysis of 124 probands in combination with available familial data increases the understanding of penetrance and variability of mutations in DICER1 and the effect of these mutations on phenotype. Their identification of additional mosaic, germline and sporadic mutations helps to further elucidate phenotypic differences between these groups and provides insight into disease pathogenesis. Their proposed temporal model of mutation acquisition correlating to disease presentation is supported by the data. However, the following points need clarification:

**Abstract:**

On page two the authors state: “A final category of patients lack predisposing germline or mosaic mutations and have disease limited to a single PPB tumor bearing tumor-specific RNase IIIb and LOF mutations.”

Cases with a single PPB tumor should not be included under the umbrella of the DICER1 syndrome. A single tumor bearing a causative mutation does not merit inclusion as syndromic, which requires wide spread distribution of mutations in that same gene. This classification is not accurate as these patients simply represent a sporadic neoplasm.

**Subjects and Methods:**

*Mutation Testing*

On page three the authors state: “Initial sequencing of blood and saliva DNA samples was by standard Sanger methods described previously or by a commercial laboratory (Ambry Genetics, Aliso Viejo, CA). Low-frequency variants were detected and quantified by targeted next-generation sequenc- ing (NGS) using a custom multiplex PCR panel for *DICER1 *coding regions (Ion Torrent Ampliseq, Life Technologies, Grand Island, NY, USA). NGS was performed on an Ion Torrent 318 v2 chip (ION PGM Sequencing 200 kit v2, Life Technologies) with an average of 6 samples per chip, to achieve an average depth of coverage of 3000 filtered reads.”

Did the authors use the Ion Torrent PCR panel for the analysis of the isolated tumors? This is not mentioned in the methods section. The data presented in Table 2 displays a large distribution in read numbers, which may have implications for data analysis. Can the authors provide an explanation for the wide distribution of read coverage in this table, particularly in the disparity of reads between blood and tumor samples? This should be included in the methods section.

*Annotation of sequence variants and the spectrum of possible mutations*

On page three the authors state their methods for annotating variants identified: “For variants assayed by NGS, allele frequencies were calculated from filtered read counts. The SIFT algorithm was used to assess potential significance of novel missense mutations.”

It would be helpful, and strengthen the author’s argument that these mutations are pathogenic, to include an analysis of the frequency of loss of function (LOF) and hotspot mutations in the population by determining their minor allele frequency (i.e. using ExAC or 1000 genomes).  

*NanoString genomic copy number assay*

On page four the authors state: *“*Genomic DNA was fragmented and hybridized using the nCounter Prep Station, and hybridization signals quantified using the nCounter Digital Analyzer, according to NanoString’s recommendations.”

It is stated in this section that hybridization signals were quantified. However, in table two many of the second hit LOF mutations are simple listed as “allele loss” and not quantified. Can the specific allele region and its quantification be provided as a percentage of allele loss abundance (as was done in table S8 for “Informative SNP” in cases 91,111,112)? Without this data it cannot be determined why the hot spot variant and the allele loss are unequally distributed (cases 104 and 105). Furthermore this would confirm the tumor purity estimates provided as normal cells should not have loss of the second allele.  As tumor purity increases so should percentage of allele loss if these mutations are in fact required for tumor formation. As the table reads now it is implied that the second allele loss is complete (50%) in the tumors where it was observed. If this is not the case we ask that the loss be quantified and included, otherwise the “-” should be replaced with “NM” (not measured).

Were any positive controls run to confirm the ability to specifically detect copy number events using the Nanostring assay?  For example, isolating DNA from preserved tumor samples often yields sheared fragments varying in size, which may hinder probe hybridization across fragments. This may yield false positive allele loss results.

**Results:**

*Most predisposing DICER1 mutations are inherited loss of function (LOF) mutations*

On page four the authors state: “Our overall approach to detecting and categorizing predisposing DICER1 mutations in PPB children is shown schematically in Figure 1. We identified germline, heterozygous DICER1 mutations in 90 of the 124 probands in our cohort (72.6%; Table 1, Table S3).”

Are the identified LOF variants observed in the general population (ExAC, 1000 Genomes)?

On page four the authors state: “In all, 84 of 90 germline *DICER1 *mutations discovered in patients (93%) truncate the open reading frame before the end of the critical RNase IIIb domain, and are thus predicted to result in complete loss of DICER1 protein function even if the message escapes nonsense-mediated decay.”

There is no mention of potential alternative splice isoforms of DICER1, which may be translated despite the presence of early stop and/or frameshift mutations. This is an oversight especially as there is an emerging role for a specific splice variant DICER1e (a splice variant composed of only the RNase IIIa, IIIb and dsRBD domains) in neoplasms. This isoform may utilize a distinct promoter as has been observed for the glucocorticoid receptor gene ( Russcher *et al.*, 2007) and not rely on faithful sequence integrity of upstream exons. Two independent reports ( Cantini *et al.*, 2014 and Hinkal *et al.,* 2011) have shown increased DICER1e isoforms in oral cancer cells and breast cancer cells respectively. This may be an important factor in discerning potential sub categories of LOF mutations. If in fact DICER1e plays a pathogenic role it is possible that alleles bearing early stop and/or frameshift mutations upstream of the RNaseIIIb domain, which in this study account for 93% of the mutations, are still able to code for this isoform and contribute to disease. The mechanism for oncogenesis may not require a true loss of function first hit but the presence of a modified isoform which in combination with a hot spot mutation would lead to a neomorphic phenotype associated with tumor formation.  The authors should acknowledge this possibility in the manuscript. 

*Approximately 10% of predisposing DICER1 mutations are mosaic rather than germline* 

On page six the authors present table 1 “Clinical and Pathologic Features by Predisposing DICER1 Mutation Category.”

In table 1 there is a single case reported of a germline LOF mutation and a Wilms tumor (WT). Could the authors speculate on the rarity of WT in their large cohort given the described association of both single hot spot and biallelic mutations in DICER1 with this tumor type ( Klein *et al.*, 2014; Wu *et al.*, 2013)?

On page eight the authors state: “NGS of tumor tissues from these children identified somatic LOF mutations or allele loss in some but not all specimens, with the caveat that allele loss can be difficult to detect in specimens with low tumor purity ( *i.e.*, PPB Type Ir, CN and NCMH).”

If the second hit LOF mutation is indeed a “driver” mutation of neoplasm one would expect those mutations to occur early in tumor formation and then clonally expand and be present in a majority of tumor cells. The very difficulty to detect these second hit LOF mutations argues that these tumors are in fact genetically heterogeneous; suggesting that these second hit mutations may represent passenger or modifying mutations but not drivers. The caveat mentioned supports both the authors’ and the alternative hypotheses and this should be included as a possible mechanism of pathogenesis.

On page eight the authors state: “Four of the five children with mosaic *DICER1 *hotspot mutations presented with cystic PPB (type I/IR) rather than sarcomatous disease (type II or type III) and all five have survived to date.”

It is surprising that none of the mosaic hot spot cases present with PPB type II/III compared to two thirds of the germline LOF cases. This is not consistent with the more complicated clinical course and numerous neoplasms observed in the mosaic cases. Is it possible that the distribution in the tissue ultimately dictates the severity of the PPB? The authors should discuss as part of their disease model why the mosaic cases have a more complex clinical course while having more benign lung pathology.

On page eight the authors state: “Though all five hotspot mosaic children are alive, their clinical experiences have been complicated and arduous (Figure 3).”

As the authors state, cases with mosaic DICER1 hot spot mutations present with a complex clinical course. Therefore, including a more detailed clinical description of the five mosaic cases, specifically paying attention to their phenotype, would strengthen this statement. For example, including growth parameters as well as developmental and physical exam findings may help to define this subgroup of the DICER1 syndrome. On page 14 of the supplement a footnote to table S9 mentions “This data set is limited, pursuant to concerns for potential identification of study participants based on particular combinations of clinical and pathologic features. Qualified investigators with specific questions about the study not answered by the data in these tables are invited to contact the International PPB Registry. The Registry will try to accommodate requests for additional data while preserving protected health information.” This concern may be the very reason why more detailed phenotypic data is lacking. However this limits the ability for the reader to draw genotype-phenotype correlations and to compare these cases to those already reported in the literature.  We request that at least common clinical findings shared by mosaic cases (if any) are presented in a table without reference to individual cases. This should limit the risk of potential identification of study participants.

*Table 2*

On page eight the authors state: “We categorized a *DICER1 *mutation detected by NGS as mosaic when the following criteria were met: *i. *The mutation was evidently not a constitutional, germline allele because it was present at sub-heterozygous frequency (arbitrarily taken as below 35% of reads) in peripheral blood and/or other normal tissue samples. *ii. *The mutation was evidently not specific to a tumor, because the same mutant allele was detected in one or more normal, non-neoplastic tissue samples, OR, the same mutant allele was detected in multiple primary tumors arising in different organs.”

It is possible that low abundance mutations detected in blood are not present in a blood cell lineage but may represent metastatic disease (for example in case 102 where the brain tumor is a metastatic event that originated in the lung PPB)? Furthermore cases 102, 103, 104, and 105 have very low mutation abundance in blood with cases 103-105 carrying less than 0.3%. These low numbers are perhaps evidence of unrecognized metastatic disease in these patients, the detection of which has been previously described ( Haber and  Velculescu, 2014). Testing of more “normal” tissues is needed for fulfilling the mosaicism criteria as proposed by the authors. It is more likely that the cases in table 2 have lower levels of mosaicism limited to a small number of tissues in contrast to other cases with more widely distributed hot spot mutations (Klein *et al.*, 2014). Additional phenotypic clinical data for these cases (as requested above) is needed to properly make a comparison.  

Tumor purity is very low in mosaic cases samples (Table 2), and this is used as an argument for why second hit mutations are lower in abundance. However, it is also possible that the second hit mutation is unequally distributed throughout the tumor and in fact absent from some regions of the tumor. This possibility should be mentioned in the manuscript.

*Tumor-specific, biallelic DICER1 mutations account for about 10% of PPB cases*

On page eight the authors state: “In twelve children, we identified biallelic *DICER1 *mutations present at high allele frequencies in a PPB tumor, but not detectable in blood even with the benefit of high-depth NGS (Table S8).”

These cases likely represent sporadic neoplasm mutations. We question if these cases should be classified as having the “DICER1 syndrome.” A more clear distinction between isolated PPB and the “DICER1 Syndrome” should be included.

*Currently unresolved cases*

On page eight the authors state: “Twelve PPB probands in our cohort are negative for predisposing *DICER1 *mutations detectable in blood DNA by Sanger sequencing or NGS of coding exons.”

The authors include an additional 12 unresolved cases (Table S9). We suggest that a potential etiology for cases in which DICER1 mutations are absent would be caused by mutations in DROSHA or other genes involved in the microRNA processing pathway. It has been established that at least in the pathogenesis of sporadic Wilms tumor RNaseIIIb mutations in DROSHA phenocopy those in DICER1, although possibly by distinct mechanisms (Rakheja, 2014). While the authors pursued DROSHA testing via Sanger sequencing exclusively in blood (Table S9 Footnote: “Sanger sequencing in blood DNA for DROSHA, XPO5, and the DICER1 promoter region”) this approach might have missed low abundance mosaic mutations. Furthermore, some of these “unresolved” cases (121, 123 and 124) have been identified as carrying hot spot mutations in their tumors without the presence of a second hit. This may illustrate that the hotspot mutations alone may be sufficient for tumorogenesis.

On page 5 the authors present Figure 1 “Study Design”

After reviewing this cohort we call into question the designation of “unresolved” in one case. We would like to propose that case 123 is actually mosaic due to the presence of the same hotspot mutation in two distinct disease foci. This case cannot be excluded as mosaic based on the absence of the mutation in blood as it is similar to case 105 where the sequencing from blood is not above the error threshold. Case 123 should therefore be moved to table two, and the study design/mosaic criteria amended to classify case 105 and 123 as mosaic even in the absence of the hotspot mutation in normal tissue.  The absence of a LOF second hit in the presence of a mosaic hot spot mutation should represent a distinct subset of mosaic cases and not classified as “unresolved.”

**Discussion:**

*Genotype-phenotype correlation of predisposing mutations in PPB-DICER1 syndrome*

On page ten the authors state: “All germline *DICER1 *truncating mutations are predicted to be essentially equivalent in their effect: complete loss of function in miRNA processing.”

As mentioned above this conclusion must be tempered by the possibility of an alternatively spliced variant of DICER1 that could be expressed despite a truncating mutation upstream of the RNase IIIb domain.  

On page ten the authors state: “Neomorphic RNase IIIb domain function (skewed 5p/3p miRNA production) is a recurring feature of *DICER1 *tumors, and it is plausible that loss of all wildtype RNase IIIb function is required for it to become tumorigenic.”

The statement “loss of all wildtype RNase IIIb function is required for it to become tumorigenic” does not apply to all categories of the DICER1 syndrome. From the data as it is presented in this study the only category where this can be concluded is from the sporadic tumors, which are distinct from the DICER1 syndrome. In these tumors it is clear from the data in table S8 that all hot spot mutations are accompanied by LOF mutations with corresponding abundances, which are certainly a characteristic of these aggressive lung neoplasms. However, we cannot conclude causality for the second hit mutations in all DICER1 tumors since (1) in the mosaic hot spot cases (Table 2) the observed abundance for the second LOF hit mutations is always less than the hot spot mutation and (2) there are cases of tumors in this report that lack a second hit (Case 105: NCMH, Case 123 CN and PPB). A main objection to the analysis and interpretation of these results is the lack of an explanation for the differences in mutation abundance between LOF and hot spot mutations within a tumor and the presence of tumors without a second hit. This raises questions as to whether the LOF mutations are in fact drivers of tumorigenesis or passenger mutations. Although tumor purity could be partially responsible for these inconsistencies, the data on its own does not sufficiently establish that these LOF mutations are required for tumor formation in non-sporadic tumor cases when they are not present in all neoplastic cells.

In the model as it is proposed by the authors, cases that are mosaic for RNaseIIIb mutations display no clinical findings until a second LOF mutation occurs which drives and is essential for tumor formation (Page 11, last paragraph). We believe there is the possibility for another explanation. A single RNase IIIb mutation alone could have a pro-onocogenic effect on distinct cell types at specific developmental stages. As tumorigenesis proceeds, a LOF mutation in the other allele may arise as the tumor drifts, further aggravating the 5p/3p imbalance in a sub population of tumor cells. Supporting this alternative model, cases 105, 121, 123 and 124 are reported to have neoplasms with no second hit detected. If this second hit is essential why do these tumors lack the LOF second hits?  In aggregate there are 4 mosaic cases (2 in this report and 2 in the literature), which, in combination with the absence of germline true heterozygote hot spot mutations, support the alternate model that mosaic hot spot mutations are likely pathogenic on their own and the authors should expand their model to include this alternate explanation.

**Supplemental Data**

*Table S5* *Clinical features of children with germline DICER1 mutations*

Can the authors comment on why mortality is higher in germline LOF mutation carriers than it is in the mosaic “hot spot” mutation carriers even though the latter have a more complicated clinical course? Could this be due to the association of PPB type I/IR with mosaic cases and PPB type II and III with germline LOF cases?

**Minor Changes**

1. The authors should include appropriate references to any manuscripts in which any of these PPB registry cases have been previously reported. 
2. While not essential, it would be informative to include any affected siblings for the 10 identified *de novo *LOF cases to support or refute potential germline mosaicism in the parents (Page 4)?
3. Table 1 includes a single case of a germline LOF mutation and a Wilms tumor (WT). However, little else is described about this case. Furthermore there is no mention of this case in the supplementary materials. Please include mutation analysis and additional phenotypic information for this case. 

We have read this submission. We believe that we have an appropriate level of expertise to confirm that it is of an acceptable scientific standard, however we have significant reservations, as outlined above.

# Response

**Published:** 2018-01-11

**Authors:** D. Ashley Hill

**Affiliations:** Children's National Medical Center, USA

**Author Responses in Bold to Referee Repor**t

 Julian A. Martinez-Agosto, Departments of Pediatrics and Human Genetics, David Geffen School of Medicine, University of California, Los Angeles, CA, USA 

 Steven Klein, Departments of Pediatrics and Human Genetics, David Geffen School of Medicine, University of California, Los Angeles, USA 

**Approved with Reservations**

 Brenneman et al. present an observational study on a cohort of patients with DICER1 Syndrome. The analysis of 124 probands in combination with available familial data increases the understanding of penetrance and variability of mutations in DICER1 and the effect of these mutations on phenotype. Their identification of additional mosaic, germline and sporadic mutations helps to further elucidate phenotypic differences between these groups and provides insight into disease pathogenesis. Their proposed temporal model of mutation acquisition correlating to disease presentation is supported by the data. However, the following points need clarification:

**Abstract:**

 On page two the authors state: *“A final category of patients lack predisposing germline or mosaic mutations and have disease limited to a single PPB tumor bearing tumor-specific RNase IIIb and LOF mutations.”*

 Cases with a single PPB tumor should not be included under the umbrella of the DICER1 syndrome. A single tumor bearing a causative mutation does not merit inclusion as syndromic, which requires wide spread distribution of mutations in that same gene. This classification is not accurate as these patients simply represent a sporadic neoplasm.

**Right. We don’t consider these to be cases of *DICER1* syndrome, and didn’t actually refer to them as such in the article. But we didn’t think to explicitly state the distinction between sporadic and syndromic PPB. We should have, and that has been corrected, both in the abstract and the *Results* section. This was a prospective study of PPB, so in our report we have accounted for all cases of PPB that were accrued for sequencing, whether they turned out to meet criteria for *DICER1* syndrome or not. The title of the article has also been revised to reflect our use of PPB as the basis for accrual. It may still imply to some readers that all cases being described are *DICER1* syndrome, but we can’t think of a way to avoid that without making the title absurdly long.**

**Subjects and Methods:**

***Mutation Testing***

 On page three the authors state: *“Initial sequencing of blood and saliva DNA samples was by standard Sanger methods described previously or by a commercial laboratory (Ambry Genetics, Aliso Viejo, CA). Low-frequency variants were detected and quantified by targeted next-generation sequenc- ing (NGS) using a custom multiplex PCR panel for DICER1 coding regions (Ion Torrent Ampliseq, Life Technologies, Grand Island, NY, USA). NGS was performed on an Ion Torrent 318 v2 chip (ION PGM Sequencing 200 kit v2, Life Technologies) with an average of 6 samples per chip, to achieve an average depth of coverage of 3000 filtered reads.”*

 Did the authors use the Ion Torrent PCR panel for the analysis of the isolated tumors? This is not mentioned in the methods section. The data presented in Table 2 displays a large distribution in read numbers, which may have implications for data analysis. Can the authors provide an explanation for the wide distribution of read coverage in this table, particularly in the disparity of reads between blood and tumor samples? This should be included in the methods section.

**All sequencing of tumors was done on the Ion Torrent platform, using the panel of PCR amplicons for *DICER1* coding regions detailed in Supplemental Data (Table S1). The variation in total read numbers apparent in Table 2 is related to several factors: the number of sequencing runs done for a sample, the number of reads per run that pass quality metrics, the “purity” of tumor specimens and the occurence of allele loss in some. For some blood samples, fewer samples were loaded per chip, or multiple sequencing runs were done, to get higher total read numbers and improve the sensitivity of mutation detection. This was so particularly in cases where initial Sanger sequencing of blood was negative, but we had reason to suspect a mosaic mutation might be present at low allele frequency ( *i.e.*, a mutation had already been detected in more than one tissue/tumor specimen).**

***Annotation of sequence variants and the spectrum of possible mutations***

 On page three the authors state their methods for annotating variants identified: *“For variants assayed by NGS, allele frequencies were calculated from filtered read counts. The SIFT algorithm was used to assess potential significance of novel missense mutations.”*

 It would be helpful, and strengthen the author’s argument that these mutations are pathogenic, to include an analysis of the frequency of loss of function (LOF) and hotspot mutations in the population by determining their minor allele frequency ( *i.e.,* using ExAC or 1000 genomes).

**None of the specific LOF or hotspot variants we identified in this study have been reported as germline alleles in the 1000 Genomes data base, but that’s beside the point really. We know that many of the LOF alleles in our cohort occur in people who have never been diagnosed with any form of syndromic disease, because there are many confirmed but asymptomatic carriers identified among family members in the PPB Registry. In other words, the penetrance of *DICER1* mutations in pedigrees is low. So, unfortunately, population data isn’t very helpful in establishing the pathogenicity of individual mutations.**

***NanoString genomic copy number assay***

 On page four the authors state: *“Genomic DNA was fragmented and hybridized using the nCounter Prep Station, and hybridization signals quantified using the nCounter Digital Analyzer, according to NanoString’s recommendations.”*

 It is stated in this section that hybridization signals were quantified. However, in table two many of the second hit LOF mutations are simply listed as “allele loss” and not quantified. Can the specific allele region and its quantification be provided as a percentage of allele loss abundance (as was done in table S8 for “Informative SNP” in cases 91,111,112)? Without this data it cannot be determined why the hot spot variant and the allele loss are unequally distributed (cases 104 and 105). Furthermore this would confirm the tumor purity estimates provided as normal cells should not have loss of the second allele. As tumor purity increases so should percentage of allele loss if these mutations are in fact required for tumor formation. As the table reads now it is implied that the second allele loss is complete (50%) in the tumors where it was observed. If this is not the case we ask that the loss be quantified and included, otherwise the “-” should be replaced with “NM” (not measured).

 Were any positive controls run to confirm the ability to specifically detect copy number events using the Nanostring assay?  For example, isolating DNA from preserved tumor samples often yields sheared fragments varying in size, which may hinder probe hybridization across fragments. This may yield false positive allele loss results.

**In this project, we used the NanoString Copy Number Assay only to interrogate genomic DNA extracted from blood for *DICER1* exonic deletions in a handful of cases. The *Subjects and Methods* section has been amended to make this clear. For detection of exon deletions in blood DNA, our NanoString controls consisted of blood DNA from children with normal diploid *DICER1* copy number, and DNA from one PPB tumor with three copies of *DICER1* as determined by next generation sequencing (Pugh *et al*, 2014). We did not use NanoString to evaluate *DICER1* copy number in tumors. In our limited experience, we found that even slight degradation of DNA in FFPE tumor specimens makes reliable detection of copy number changes with NanoString very challenging. Admixture of tumor and normal cells makes it more so.**

**Results**

***Most predisposing DICER1 mutations are inherited loss of function (LOF) mutations***

 On page four the authors state: *“Our overall approach to detecting and categorizing predisposing DICER1 mutations in PPB children is shown schematically in Figure 1. We identified germline, heterozygous DICER1 mutations in 90 of the 124 probands in our cohort (72.6%; Table 1, Table S3).” * 

 Are the identified LOF variants observed in the general population (ExAC, 1000 Genomes)?

**Please see our response above, in relation to *Annotation of sequence varaints.***

**On page four the authors state: *“In all, 84 of 90 germline DICER1 mutations discovered in patients (93%) truncate the open reading frame before the end of the critical RNase IIIb domain, and are thus predicted to result in complete loss of DICER1 protein function even if the message escapes nonsense-mediated decay.”***

**There is no mention of potential alternative splice isoforms of DICER1, which may be translated despite the presence of early stop and/or frameshift mutations. This is an oversight especially as there is an emerging role for a specific splice variant DICER1e (a splice variant composed of only the RNase IIIa, IIIb and dsRBD domains) in neoplasms. This isoform may utilize a distinct promoter as has been observed for the glucocorticoid receptor gene ( Russcher et al., 2007) and not rely on faithful sequence integrity of upstream exons. Two independent reports ( Cantini et al., 2014 and Hinkal et al., 2011) have shown increased DICER1e isoforms in oral cancer cells and breast cancer cells respectively. This may be an important factor in discerning potential subcategories of LOF mutations. If in fact DICER1e plays a pathogenic role it is possible that alleles bearing early stop and/or frameshift mutations upstream of the RNaseIIIb domain, which in this study account for 93% of the mutations, are still able to code for this isoform and contribute to disease. The mechanism for oncogenesis may not require a true loss of function first hit but the presence of a modified isoform which in combination with a hot spot mutation would lead to a neomorphic phenotype associated with tumor formation. The authors should acknowledge this possibility in the manuscript.**

****

**DICER1e is intriguing but still a very early story. It’s not known whether or how this isoform participates in miRNA processing or other possible DICER1 functions. No specific mechanism by which differential expression of a DICER1e protein isoform might contribute to tumorigenesis has been established. It’s also not yet known whether the *DICER1e* mRNA is an alternate splice product or an independent transcript from a different promoter. It’s not clear whether or how truncating mutations in upstream exons influence expression of the *DICER1e* mRNA. And unfortunately, our data includes no information about whether a *DICER1e* mRNA or protein was expressed in any of the tumors of patients who were included in this study. As we have nothing to support it, we have chosen not to speculate about DICER1e in this report.**

****

**But hypothetically, let’s assume that:**

***i*.) DICER1e has distinct activities that can contribute to tumorigenesis,**

***ii.*) DICER1e can be expressed regardless of mutations in upstream exons, and**

***iii.*) expression of DICER1e, rather than loss of full-length DICER1, is the important co-determinant of tumorigenesis when combined with an RNase IIIb hotspot mutation.**

****

**We then have to explain why germline mutations in exons 21-27 (n = 46, Figure 3), which presumably would inactivate DICER1e *,* seem to correlate with disease just as strongly as mutations in exons 2-18 (n = 44), which would not. Suggestions?**

***Approximately 10% of predisposing DICER1 mutations are mosaic rather than germline***

 On page six the authors present Table 1. *Clinical and Pathologic Features by Predisposing DICER1 Mutation Category*. In Table 1, there is a single case reported of a germline LOF mutation and a Wilms tumor (WT). Could the authors speculate on the rarity of WT in their large cohort given the described association of both single hot spot and biallelic mutations in DICER1 with this tumor type ( Klein et al., 2014; Wu et al., 2013)?

 In addition to the two remarkable cases of Wilms tumor with *DICER1 *mutation described in your own paper, and those in Wu *et al,* 2013, we’re aware of the cases published by Rakeja *et al,* 2014 and Torrezan *et al,* 2014.

**The low incidence of Wilms tumor in the present cohort may reflect some ascertainment bias. The criterion for inclusion in this cohort was a diagnosis of pleuropulmonary blastoma (PPB). But there have been children with other *DICER1*-associated tumors in whom PPB was not diagnosed first (or at all). Ascertainment bias might also pertain to other *DICER1*-associated tumors that are rare or absent in our cohort, *i.e.,* pituitary blastoma. That said, the frequency of Wilms tumor in this cohort roughly parallels what we have seen among PPB children and their extended families in the International PPB Registry more generally, *i.e.*, it is low (about 1%), similar to pineoblastoma and pituitary blastoma. In our experience, cystic nephroma is far more common than Wilms tumor or Wilms precursor lesions (nephrogenic rests, nephroblastomatosis) in DICER1 syndrome. Cystic nephroma can progress to primitive renal sarcoma, which has some morphologic overlap with Wilms tumor, but clearly there are also classic-morphology Wilms tumors with *DICER1 *mutations. How the genetic etiologies of these neoplasias converge or diverge is yet to be discovered.**

 On page eight the authors state: *“NGS of tumor tissues from these children identified somatic LOF mutations or allele loss in some but not all specimens, with the caveat that allele loss can be difficult to detect in specimens with low tumor purity (i.e., PPB Type Ir, CN and NCMH).”*

 If the second hit LOF mutation is indeed a “driver” mutation of neoplasm one would expect those mutations to occur early in tumor formation and then clonally expand and be present in a majority of tumor cells. The very difficulty to detect these second hit LOF mutations argues that these tumors are in fact genetically heterogeneous; suggesting that these second hit mutations may represent passenger or modifying mutations but not drivers. The caveat mentioned supports both the authors’ and the alternative hypotheses and this should be included as a possible mechanism of pathogenesis.

**The passage you cite has been revised to note that specific LOF mutations or evidence of allele loss were found in *all*tumor specimens sequenced for hotspot mosaic patients. Please see the revised Table 2. Please also see our response to Drs. Chen and Amatruda, which includes an extended discussion of how allele loss has been inferred from NGS read counts, with examples. We agree with you that at least some of the tumors described in Table 2 must be genetically heterogeneous. The tumor specimens sequenced for patient #103 are the clearest examples. We also agree that the *DICER1* LOF mutations we detected should not be considered driver mutations. The term *driver mutation* is usually reserved for gain-of-function events causing activation or overexpression of oncogenes, such as *KRAS* or *MYC,* that stimulate (“drive”) tumor cell growth and division. It’s not just a matter of labels. Continued expression of an activated oncogene is subject to positive selection during expansion of a tumor cell population. But for a tumor suppressor gene, a specific loss-of-function change in coding sequence will not always be retained by selective pressure. Even if continuing absence of expression is needed for growth after initiation of a tumor, any kind of subsequent LOF mutational event can suffice to maintain it: complete or partial deletion of the gene, translocations or inversions that disrupt the open reading frame, copy-neutral loss of heterozygosity through gene conversion or mitotic crossover, complete or partial chromosome loss, etc. Many of these events will not be detected by exon sequencing. Thus, some level of heterogeneity with regard to LOF mutations should not come as a surprise; neither should low allele frequency of a specific LOF coding sequence change, nor the absence of such changes in some tumor cell populations.**

**The question of why specific LOF mutations are detected at low allele frequencies (or not at all) in some tumors of hotspot mosaic children, and what the true significance of LOF mutations in tumorigenesis may be, has also attracted the attention of our other two reviewers, Kenneth Chen and Jim Amatruda. They also suggest an alternative hypothesis, which may be similar to what you have in mind. Please take a look at our response to Drs. Chen and Amatruda and at the revised *Discussion* section, in which we now explicitly raise the possibility of a dominant-negative effect for hotspot mutations, in some tissues at least. We look forward to discussing this further with you in the future.**

 On page eight the authors state: *“Four of the five children with mosaic DICER1 hotspot mutations presented with cystic PPB (type I/IR) rather than sarcomatous disease (type II or type III) and all five have survived to date.”*

 It is surprising that none of the mosaic hotspot cases present with PPB type II/III compared to two thirds of the germline LOF cases. This is not consistent with the more complicated clinical course and numerous neoplasms observed in the mosaic cases. Is it possible that the distribution in the tissue ultimately dictates the severity of the PPB? The authors should discuss as part of their disease model why the mosaic cases have a more complex clinical course while having more benign lung pathology.

**One of the hotspot mosaic children had both PPB type II and type Ir; this was noted in Table 1. That patient (study ID# 102) recently died of metastatic PPB. Another patient now recategorized as hotspot mosaic (study ID# 120) also had both PPB type II and type Ir. Even so, incidence of advanced PPB has been low among the hotspot mosaic children as a group, and we also found this surprising. But it’s not clear whether hotspot mosaic children actually have less aggressive lung pathology, or simply tend to get treated earlier, before it progresses. All the children in this group developed bilateral, multifocal lung cysts, and at very early ages, similarly to the two mosaic cases you described. Their early diagnosis was no doubt in part because their lung lesions, though type I or Ir, were numerous and/or large enough to cause obvious breathing difficulty. For children who have germline LOF mutations this is not always the case. They often develop only a single PPB lesion in one lung, and as a group tend to be diagnosed later (Table 1). This means the lesions have more time to progress from type I to type II or III, contributing to higher incidence of advanced PPB and metastatic disease at diagnosis and greater mortality. The possibility you suggest, that distribution of the mutation might influence not just the number of lesions but also their severity, is not one we thought of before. Nothing in our data rules that out.**

 On page eight the authors state: *“Though all five hotspot mosaic children are alive, their clinical experiences have been complicated and arduous (Figure 3).” * 

 As the authors state, cases with mosaic *DICER1* hot spot mutations present with a complex clinical course. Therefore, including a more detailed clinical description of the five mosaic cases, specifically paying attention to their phenotype, would strengthen this statement. For example, including growth parameters as well as developmental and physical exam findings may help to define this subgroup of the *DICER1* syndrome. A footnote to table S9 mentions *“This data set is limited, pursuant to concerns for potential identification of study participants based on particular combinations of clinical and pathologic features. Qualified investigators with specific questions about the study not answered by the data in these tables are invited to contact the International PPB Registry. The Registry will try to accommodate requests for additional data while preserving protected health information.”* This concern may be the very reason why more detailed phenotypic data is lacking. However this limits the ability for the reader to draw genotype-phenotype correlations and to compare these cases to those already reported in the literature. We request that at least common clinical findings shared by mosaic cases (if any) are presented in a table without reference to individual cases. This should limit the risk of potential identification of study participants.

**All the shared characteristics of the hotspot mosaic children that we know of have been presented in the figures and tables, and further described in the text. Figure 3 presents all syndromic disease and age at each diagnosis individually for each patient. Table 1 provides a summary of all syndromic disease for the hotspot mosaic children as a group, and statistical comparisons to other categories. We understand your particular interest in body growth metrics and developmental milestones, and regret we cannot furnish them. But we don’t have detailed physical exam information for the seven children ultimately identified as hotspot mosaic. This was a prospective study conducted at multiple centers. Participating clinicians didn’t report that information for all probands over the course of this study, probably because they weren’t asked to. In the past, abnormalities of growth and developmental delays had generally not been perceived as features of familial PPB/DICER1 syndrome, and the mosaic hotspot phenomenon was only identified in retrospect.**

**Table 2**

 On page eight the authors state: *“We categorized a DICER1 mutation detected by NGS as mosaic when the following criteria were met: i. The mutation was evidently not a constitutional, germline allele because it was present at sub-heterozygous frequency (arbitrarily taken as below 35% of reads) in peripheral blood and/or other normal tissue samples. ii. The mutation was evidently not specific to a tumor, because the same mutant allele was detected in one or more normal, non-neoplastic tissue samples, OR, the same mutant allele was detected in multiple primary tumors arising in different organs.”*

 It is possible that low abundance mutations detected in blood are not present in a blood cell lineage but may represent metastatic disease (for example in case 102 where the brain tumor is a metastatic event that originated in the lung PPB)? Furthermore cases 102, 103, 104, and 105 have very low mutation abundance in blood with cases 103-105 carrying less than 0.3%. These low numbers are perhaps evidence of unrecognized metastatic disease in these patients, the detection of which has been previously described ( Haber and  Velculescu, 2014). Testing of more “normal” tissues is needed for fulfilling the mosaicism criteria as proposed by the authors. It is more likely that the cases in table 2 have lower levels of mosaicism limited to a small number of tissues in contrast to other cases with more widely distributed hot spot mutations (Klein et al., 2014). Additional phenotypic clinical data for these cases (as requested above) is needed to properly make a comparison.

**It’s conceivable the very small numbers of hotspot mutation reads in blood samples from patients 103, 104 and 105 were from tumor cells shed into the circulation (the 4.6% frequency in patient 102 seems too high for that). Regardless of their origin, we didn’t consider those blood reads reliable evidence of mosaicism, and based no conclusions upon them. Our conclusion in favor of mosaicism in patients 103, 104 and 105 (and the patient recategorized in revision, ID# 123) is based upon presence of the same hotspot mutation in multiple primary tumors and/or non-neoplastic tissues. Certainly it would help to have more normal tissue specimens to sequence, but none were available and we can hardly request medically unnecessary biopsies to improve our data set. We stand by our conclusion of mosaicism, because it seems to us far more plausible than the only other  explanation possible: that the same somatic hotspot mutation occurred independently in two to five different tissue and/or tumor sites.**

 Tumor purity is very low in mosaic cases samples (Table 2), and this is used as an argument for why second hit mutations are lower in abundance. However, it is also possible that the second hit mutation is unequally distributed throughout the tumor and in fact absent from some regions of the tumor. This possibility should be mentioned in the manuscript.

**Yes, tumor purity was generally lower in specimens from hotspot mosaic children than other patient categories, probably due at least in part to the greater proportion of non-PPB tumors encountered. Some syndromic lesions such as PPB type I/Ir, cystic nephroma and juvenile-type polyps of the small intestine have complicated histo-pathological features, comprising mixtures of neoplastic and non-neoplastic cell types.**

**We agree that at least some of the LOF mutations reported in Table 2 must be unevenly distributed in the tumors specimens sequenced, particularly for study ID# 103. We didn’t cite low tumor purity as a reason for differences between hotspot and LOF allele frequencies, but it does make determinations of allele loss less certain, and the two things are related, as discussed in our responses above.**

***Tumor-specific, biallelic DICER1 mutations account for about 10% of PPB cases***

 On page eight the authors state: “ *In twelve* *children, we identified biallelic DICER1 mutations present at high allele frequencies in a PPB tumor, but not detectable in blood even with the benefit of high-depth NGS (Table S8).*” These cases likely represent sporadic neoplasm mutations. We question if these cases should be classified as having the “DICER1 syndrome.” A more clear distinction between isolated PPB and the “DICER1 Syndrome” should be included.

**As noted above in relation to the abstract, we concur that sporadic PPB should be distinguished from DICER1 syndrome. The paragraph you cite has been revised to make this clear.**

***Currently unresolved cases***

 On page eight the authors state: *“Twelve PPB probands in our cohort are negative for predisposing DICER1 mutations detectable in blood DNA by Sanger sequencing or NGS of coding exons.”*

 The authors include an additional 12 unresolved cases (Table S9). We suggest that a potential etiology for cases in which *DICER1* mutations are absent would be caused by mutations in *DROSHA* or other genes involved in the microRNA processing pathway. It has been established that at least in the pathogenesis of sporadic Wilms tumor, RNaseIIIb mutations in DROSHA phenocopy those in DICER1, although possibly by distinct mechanisms (Rakheja, 2014). While the authors pursued DROSHA testing via Sanger sequencing exclusively in blood (Table S9 Footnote: “Sanger sequencing in blood DNA for DROSHA, XPO5, and the DICER1 promoter region”) this approach might have missed low abundance mosaic mutations. Furthermore, some of these “unresolved” cases (121, 123 and 124) have been identified as carrying hot spot mutations in their tumors without the presence of a second hit. This may illustrate that the hotspot mutations alone may be sufficient for tumorogenesis. After reviewing this cohort we call into question the designation of “unresolved” in one case. We would like to propose that case 123 is actually mosaic due to the presence of the same hotspot mutation in two distinct disease foci. This case cannot be excluded as mosaic based on the absence of the mutation in blood as it is similar to case 105 where the sequencing from blood is not above the error threshold. Case 123 should therefore be moved to table two, and the study design/mosaic criteria amended to classify case 105 and 123 as mosaic even in the absence of the hotspot mutation in normal tissue. The absence of a LOF second hit in the presence of a mosaic hot spot mutation should represent a distinct subset of mosaic cases and not classified as “unresolved.”

**The 12 cases that were detailed in supplemental Table S9 *are* the 12 cases mentioned in the sentence you quote, not additional. The sentence has been revised to make that clear. Table S9 has become Table S10 in the revised paper, and now presents only 10 unresolved cases.**

**With regard to other genetic etiologies, yes, the limited genomic sequencing we did might easily have missed many kinds of mutations in *DROSHA* or *XPO5.* And there are additional candidate genes we didn’t look at. To us it seems quite possible that mutations in other genes of the miRNA processing pathway might phenocopy *DICER1* mutation in PPB or additional neoplasms besides Wilms tumor. Perhaps it’s just a matter of time before solid evidence of that turns up.**

**Your assessment of patient 123 is correct. Since the original submission of this report, additional sequencing results have given us the confidence to call hotspot mosaicism, and patient 123 now appears in Table 2 and Figure 3. We found no LOF mutation by Ion Torrent sequencing of the coding exons, but the hotspot allele frequencies obtained from read counts suggest loss of the second *DICER1* allele in the two tumor specimens sequenced. Please see the extended discussion of how allele loss was inferred in our response to Drs. Chen and Amatruda.**

**After we posted this report, the Foulkes lab published an overlapping one describing three of the same *DICER1 * patients: study ID#s 102, 105 and 120 (Patients 1, 2 and 4, respectively, in their report). They propose that patient 120 is also hotspot mosaic, but with very limited tissue distribution of the hotspot mutation, possibly confined to the lungs. We concur. But take a look and draw your own conclusions: de Kock *et al*, Journal of Medical Genetics, doi: 10.1136/jmedgenet-2015-103428. Patient 120 also now appears in Table 2 and Figure 3.**

**Discussion**

***Genotype-phenotype correlation of predisposing mutations in PPB-DICER1 syndrome***

*On page ten the authors state: “All germline DICER1 truncating mutations are predicted to be essentially equivalent in their effect: complete loss of function in miRNA processing.”*

 As mentioned above this conclusion must be tempered by the possibility of an alternatively spliced variant of DICER1 that could be expressed despite a truncating mutation upstream of the RNase IIIb domain.

 On page ten the authors state: *“Neomorphic RNase IIIb domain function (skewed 5p/3p miRNA production) is a recurring feature of DICER1 tumors, and it is plausible that loss of all wildtype RNase IIIb function is required for it to become tumorigenic.”*

 The statement “loss of all wildtype RNase IIIb function is required for it to become tumorigenic” does not apply to all categories of the DICER1 syndrome. From the data as it is presented in this study the only category where this can be concluded is from the sporadic tumors, which are distinct from the DICER1 syndrome. In these tumors it is clear from the data in table S8 that all hot spot mutations are accompanied by LOF mutations with corresponding abundances, which are certainly a characteristic of these aggressive lung neoplasms. However, we cannot conclude causality for the second hit mutations in all DICER1 tumors since (1) in the mosaic hot spot cases (Table 2) the observed abundance for the second LOF hit mutations is always less than the hot spot mutation and (2) there are cases of tumors in this report that lack a second hit (Case 105: NCMH, Case 123 CN and PPB). A main objection to the analysis and interpretation of these results is the lack of an explanation for the differences in mutation abundance between LOF and hot spot mutations within a tumor and the presence of tumors without a second hit. This raises questions as to whether the LOF mutations are in fact drivers of tumorigenesis or passenger mutations. Although tumor purity could be partially responsible for these inconsistencies, the data on its own does not sufficiently establish that these LOF mutations are required for tumor formation in non-sporadic tumor cases when they are not present in all neoplastic cells.

 In the model as it is proposed by the authors, cases that are mosaic for RNaseIIIb mutations display no clinical findings until a second LOF mutation occurs which drives and is essential for tumor formation (Page 11, last paragraph). We believe there is the possibility for another explanation. A single RNase IIIb mutation alone could have a pro-onocogenic effect on distinct cell types at specific developmental stages. As tumorigenesis proceeds, a LOF mutation in the other allele may arise as the tumor drifts, further aggravating the 5p/3p imbalance in a sub population of tumor cells. Supporting this alternative model, cases 105, 121, 123 and 124 are reported to have neoplasms with no second hit detected. If this second hit is essential why do these tumors lack the LOF second hits? In aggregate there are 4 mosaic cases (2 in this report and 2 in the literature), which, in combination with the absence of germline true heterozygote hot spot mutations, support the alternate model that mosaic hot spot mutations are likely pathogenic on their own and the authors should expand their model to include this alternate explanation.

**As mentioned above, we have revised the *Discussion* to note the model we present has apparent exceptions, and that loss of wild-type DICER1 function may not to be required in order for a hotspot mutation to instigate tumor formation in all tissues or circumstances. We point out the need to consider an alternative model, in which RNase IIIb-mutant DICER1 exerts a dominant-negative effect over the wild-type protein. We thank all of our reviewers for encouraging us to broach this possibility. At the time we first submitted the paper, we had decided against any mention of a dominant-negative model in our *Discussion,* because it seemed too speculative and we had no data of our own to directly support it. We also favor the possibility, suggested by Drs. Amatruda and Chen, that *DICER1* hotspot mutations may introduce a genome stability defect, by compromising DNA repair through as-yet-unidentified mechaisms, but chose not to mention that in the paper either, for the same reasons. But despite our general agreement, we must take exception to some of the ideas you express above.**

****

**Regarding the model we described, in which both a neomorphic *DICER1* hotspot mutation and loss of wild-type *DICER1* function are usually required to promote tumorigenesis, you declare that “ *…the only category where this can be concluded is from the sporadic tumors, which are distinct from the DICER1 syndrome.”***

****

**This is simply not the case. Please consider again the features of germline mutation patients. All have LOF mutations, either inherited or *de novo, *present at heterozygous frequency in blood or other normal tissues sampled and presumed to be present in all cells of their bodies. In all PPBs and in nearly every other tumor of these patients from which DNA of reasonable quality has been recovered and sequenced, a hotspot missense mutation was also discovered (the most conspicuous exception being the series of pineoblastomas described by the Foulkes lab, in which DICER1 function appears to have been lost entirely). If it were true that *DICER1*-associated tumorigenesis in the lung and most other vulnerable tissues does not usually require loss of wild-type DICER1 function, there would be no familial PPB/DICER1 syndrome. Similarly, children with mosaic LOF mutations would not have emerged as a category of PPB patients if LOF mutations did not impart susceptibility. The sporadic cases, in which both a hotspot mutation and LOF mutation or allele loss are confined to a single tumor, can be viewed as the exceptions that prove the rule. If concurrent loss of wild type DICER1 function is not typically necessary for a hotspot missense mutation to instigate tumorigenesis, why do they occur together even in non-syndromic PPB?**

****

**Regarding sequencing results in children with mosaic hotspot mutations: You interpret low allele frequencies of LOF mutations found in some tumor specimens, and failure to detect LOF mutations in some tumor specimens, as positive evidence that loss of wild type DICER1 function was not neccesary for tumorigenesis. But it is not positive evidence for anything beyond the inherent limitations of exon sequencing. You implicitly recognize a role for genomic instability when you suggest that LOF mutations present at low allele frequency may be only passenger mutations. Surely you can accept that ongoing genomic instability in tumor cell populations might also manifest as allele loss? The heart of the problem is this: failure to discover a specific LOF mutation in a tumor by *DICER1* exon sequencing does not establish that wild type DICER1 protein is still being expressed. If there is ever to be confirmation of our shared hypothesis that a *DICER1* hotspot mutation can be sufficient to cause tumorigenesis in some settings, it will take something more than arguments based on LOF allele frequencies.**

***Table S5. Clinical features of children with germline DICER1 mutations***

 Can the authors comment on why mortality is higher in germline LOF mutation carriers than it is in the mosaic “hotspot” mutation carriers even though the latter have a more complicated clinical course? Could this be due to the association of PPB type I/IR with mosaic cases and PPB type II and III with germline LOF cases?

**Please see our response, above, to your queries about the section of *Results* describing hotspot mosaic children. As mentioned, two of the (now seven) patients identified as hotspot mosaic had PPB type II, and one has since died of metastatic disease. But for other reasons as well, it’s not clear to us whether mortality actually is lower in the mosaic hotspot group, or whether the apparent association of mosaic hotspot mutations with PPB types I/IR is real. Consider:**

- **The hotspot mosaic children presented earlier in life for medical care. Mean age at diagnosis was less than one year. Early presentation and treatment limited time for disease progression.**
- **For other categories, there was almost certainly an ascertainment bias toward more aggressive PPB types. Many patients accrued to this study came to the attention of the PPB Registry through appeals from clinicians for advice on treating advanced PPB. Among germline LOF mutation carriers, the largest group of PPB patients by far, this typically means PPB already progressed to type II or III at the time of diagnosis.**
- **PPB type Ir (non-progressed) is likely under-diagnosed among germline mutation carriers. These are often single cystic lesions which, if small, may not cause breathing problems serious enough to elicit medical attention, and would be discovered only if thoracic imaging is done for another reason.**

**What we can say with confidence is that mortality is higher among children presenting with PPB type II or III, as compared to PPB type I/Ir, regardless of genetic etiology.**

**Minor Changes**

 The authors should include appropriate references to any manuscripts in which any of these PPB registry cases have been previously reported.

**The reference list includes most previous publications concerning PPB registry patients in which any of the present authors participated. This study surveyed the medical records of 124 patients as well as information about most of their parents and numerous additional family members. Many of these individuals have been reported in multiple publications over the last twenty years or so, and not always with our participation. We will not undertake a comprehensive tabulation of all publications that mention any patient or family member included in this study. But if you have a specific interest in particular cases, please contact us directly, and we will do our best to help.**

 While not essential, it would be informative to include any affected siblings for the 10 identified de novo LOF cases to support or refute potential germline mosaicism in the parents (Page 4)?

**There were no siblings or other family members identified with syndromic disease in the ten cases of *de novo* germline mutation. This information has been added to the text and revised supplemental Table S6. *Clinical features of children with germline DICER1 LOF mutations.* The *de novo* cases are those with a ‘0’ in the right-most column, indicating that both parents tested negative for the proband mutation.**

 Table 1 includes a single case of a germline LOF mutation and a Wilms tumor (WT). However, little else is described about this case. Furthermore there is no mention of this case in the supplementary materials. Please include mutation analysis and additional phenotypic information for this case.

**It should have been indicated in Table S6 – thank you for catching the omission. The patient with a Wilms tumor is study ID# 108. The germline *DICER1* mutation is c.1752+1delG, at the 5’ splice-site of intron 10. Skipping of exon 10 would cause in-frame deletion of 81 codons (V504 to K584) between the helicase and PAZ domains, the functional consequences of which are unknown. Actual effects of this mutation on splicing have not been examined. Syndromic disease in this male patient was confined to PPB type Ir and the Wilms tumor, which were diagnosed at age 36 months.The Wilms tumor was not available for sequencing, so we don’t know whether or what *DICER1* mutations were present. The patient is living. Two family members had syndromic conditions, but have not been screened for mutation. This information is now included in revised supplemental Tables S4 and S6.**
